# Tuneable poration: host defense peptides as sequence probes for antimicrobial mechanisms

**DOI:** 10.1038/s41598-018-33289-y

**Published:** 2018-10-08

**Authors:** Marc-Philipp Pfeil, Alice L. B. Pyne, Valeria Losasso, Jascindra Ravi, Baptiste Lamarre, Nilofar Faruqui, Hasan Alkassem, Katharine Hammond, Peter J. Judge, Martyn Winn, Glenn J. Martyna, Jason Crain, Anthony Watts, Bart W. Hoogenboom, Maxim G. Ryadnov

**Affiliations:** 1 National Physical Laboratory, Hampton Road, Teddington, TW11 0LW UK; 2 Department of Biochemistry, University of Oxford, Oxford, OX1 3QU UK; 3 London Centre for Nanotechnology, University College London, London, WC1H 0AH UK; 4 STFC Daresbury Laboratory, Daresbury, Warrington, WA4 4AD UK; 5 Department of Biochemical Engineering, University College London, London, WC1E 6BT UK; 6 IBM Research, Yorktown Heights, NY 10598 USA; 7 Department of Physics and Astronomy, University College London, London, WC1E 6BT UK; 8 Present Address: Department of Microbiology, Harvard Medical School, Boston, MA 02115 USA

## Abstract

The spread of antimicrobial resistance stimulates discovery strategies that place emphasis on mechanisms circumventing the drawbacks of traditional antibiotics and on agents that hit multiple targets. Host defense peptides (HDPs) are promising candidates in this regard. Here we demonstrate that a given HDP sequence intrinsically encodes for tuneable mechanisms of membrane disruption. Using an archetypal HDP (cecropin B) we show that subtle structural alterations convert antimicrobial mechanisms from native carpet-like scenarios to poration and non-porating membrane exfoliation. Such distinct mechanisms, studied using low- and high-resolution spectroscopy, nanoscale imaging and molecular dynamics simulations, all maintain strong antimicrobial effects, albeit with diminished activity against pathogens resistant to HDPs. The strategy offers an effective search paradigm for the sequence probing of discrete antimicrobial mechanisms within a single HDP.

## Introduction

Antimicrobial resistance challenges our ability to treat infections. Traditional approaches that rely on the inhibition of intracellular processes or cell wall synthesis with antibiotics of microbial origin contribute to the increasing number of resistant microorganisms^1^. Newly discovered, but similar compounds are subject to the same barriers of low-cost resistance mechanisms, impermeable membranes, dormant and persistent infections^1–3^. Alternative therapies require modes of action that lack such shortcomings^4^, and are consistent with the increasing use of membrane-active antibiotics such as polymyxins^5^. Host defense peptides (HDPs) are promising candidates^6^. They constitute a major part of cell-free immunity and are evolutionarily conserved. Therefore, developing widespread resistance against them is a formidable challenge for bacteria^7^. HDPs are typically cationic, readily engage with intracellular targets and favor attack on negatively charged microbial membranes of both growing and dormant bacteria, which renders them multi-target and hence generic antimicrobials^8,9^. In membranes the peptides fold as amphipathic α-helices or β-sheets that assemble into carpet-like structures, transmembrane pores or monolayer pits. Different modes of action manifest in different killing kinetics and may link to the phenotypic specificity of HDPs to bacteria^10^. Strikingly, HDP sequences are very diverse^6–10^. Apart from cationic and hydrophobic residues that are common for HDPs, amino-acid residues and motifs that are not typical for all HDPs can be incorporated to provide distinct functions. For example, terminal tryptophans are often used to anchor to membranes, and arginines are preferred over lysines for tighter electrostatic interactions in the upper leaflet of the bilayer^11^. Helix-disrupting glycine zipper motifs, G(X)_n_G, where X is any residue and n = 3–6, help control transmembrane peptide oligomerisation and specify phosphate binding as a function of responsive folding^12,13^. All these features define the shape of the folded structure, i.e. straight or kinked helix, its orientation relative to the membrane normal, i.e. parallel, perpendicular or tilted, and eventually the rate and extent of pore or carpet formation^14–18^.

Implicit correlations for membrane-disrupting mechanisms have been demonstrated for unrelated sequences. Biological activities of peptides from the same family are also compared, but do not necessarily expose distinctive membrane-disruption patterns for different family members or sequence mutants. We reason here that an HDP sequence is an intrinsic probe that encodes for different and tailorable modes of membrane disruption. Our rationale accepts membrane disruption as the primary cause of cell death^6–9^, which, consequently, puts an emphasis on two main factors:

Firstly, microbial membranes are phospholipid bilayers with a relatively universal thickness of 3–4 nm^19–21^. Gram negative bacteria comprise two bilayers, inner and outer, which are separated by a thin and porous peptidoglycan layer of 4 nm^22^. Following peptide attack on the outer membrane, the layer facilitates HDPs, to which it has high affinity, to reach the cytoplasmic membrane^23^. Gram positive membranes have only one membrane that is decorated with a thicker peptidoglycan, which may induce peptides to fold before they reach the phospholipid bilayer^24^. HDPs do not necessarily differentiate between Gram positive and Gram negative membranes, but their activity may appear variable due to the phenotypic tolerance of bacterial cells and their inherent ability to oligomerize^10,25^. Most HDPs are relatively short sequences, <25 amino-acid residues, whereas longer HDPs, <50 residues, tend to incorporate glycine zipper motifs or glycine and proline residues to regulate membrane-responsive folding^25–28^.

Secondly, shorter peptides can form conformations capable of fully inserting into microbial bilayers. An amino-acid residue in a β-strand and an α-helix would span 0.3 nm and 0.15 nm, respectively, which allows for comparably strong antimicrobial sequences of 11–21 residues in length. Longer HDPs can incorporate sub-domains to anchor to membranes, adjust to membrane curvature, thin and micellise the membranes or intercalate in the bilayer at an angle bending the bilayer pores into toroidal structures^29–31^. Synthetic sequences composed of only cationic and hydrophobic residues can be hemolytic and fold in solution via interfacing hydrophobic faces^32^. This is also common for naturally occurring HDPs including human cathelicidins whose activity varies due to their tendency to form helical bundles that propagate into filament-like structures^33^ or highly hemolytic melittins that self-regulate pore formation^34^. Effective HDPs balance cationic and hydrophobic faces to an extent ensuring a selective binding to microbial membranes. This is in contrast to cytotoxic venom or neurotoxin peptides (e.g. melittin, pardaxin) that fail to differentiate between zwitterionic mammalian and anionic microbial lipids, which obviates their use as antibiotics^35,36^.

**Figure 1:**
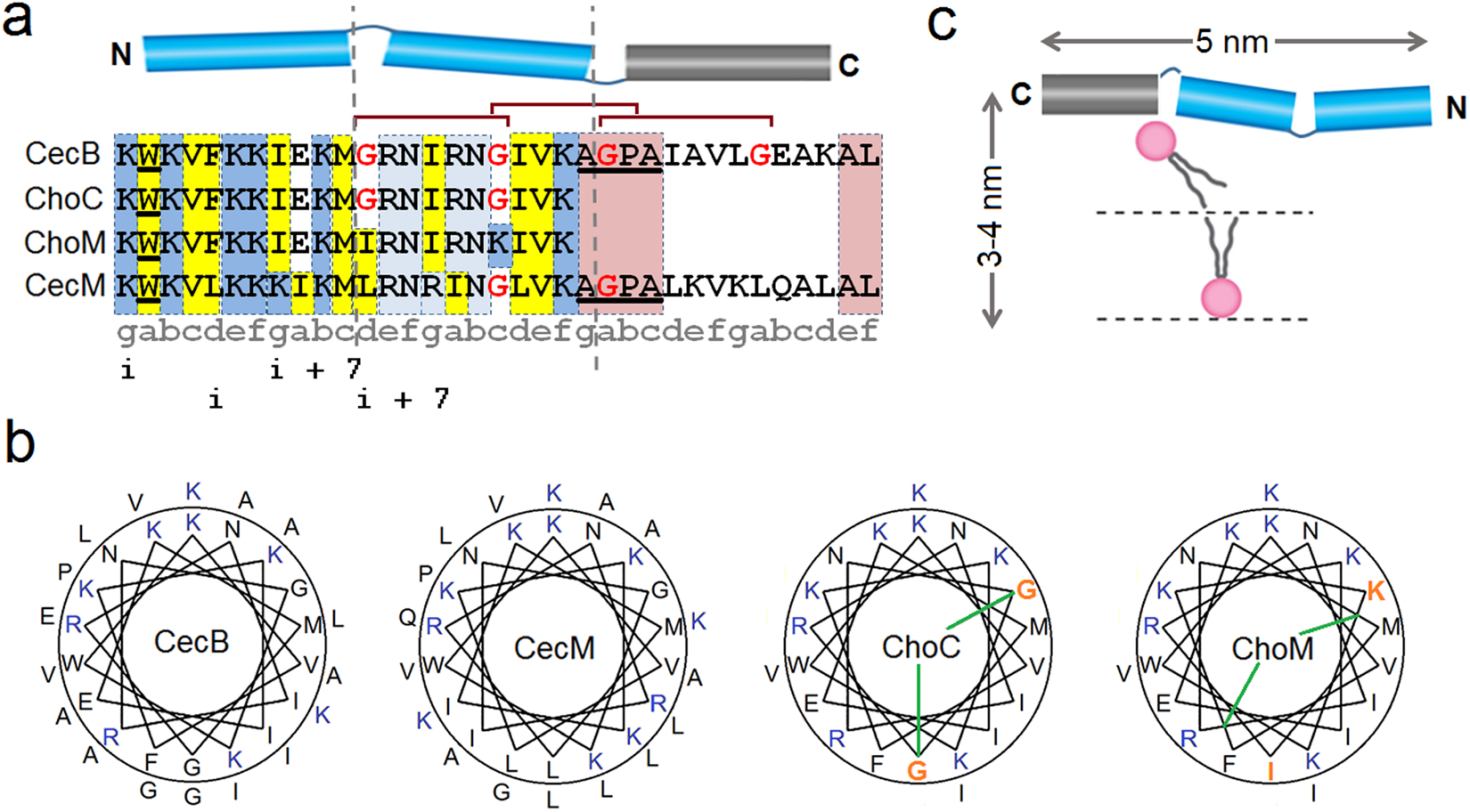
Cecropin sequence probes. Peptide sequences, linear (**a**) and configured on helical wheels (**b**). Blue and grey cylinders denote helical N-terminal (blue) and C-terminal (grey) domains. Lysines are in blue, arginines and neutral polar residues are in light blue, relative identities of hydrophobic residues are in yellow. Glycine residues are in red and glycine zippers are highlighted by overarching horizontal brown brackets. Glycine residues in ChoC and their replacements in ChoM are in orange in the helical wheels. Green lines in the helical wheels of ChoC and ChoM indicate polar angles. The AGPA hinge and the W2 residue are underlined. A coiled-coil designation, *gabcdef*, is shown along the CecM sequence. Heptad repeats are shown underneath. Only two *i, i* + 7 pairs are given for clarity. (**c**) A schematic representation of a carpet-like mechanism by CecB oriented flat on a phospholipid bilayer. For clarity, only one phospholipid per leaflet is shown (aliphatic chains in grey, headgroups in pink).

Together these factors suggest that the length and composition of a given sequence act in an interplay that discriminates and enables different modes of membrane disruption. Since HDPs from one family have little sequence similarity to other families, each family is likely to have evolved independently^26,36^. Therefore, we explore here the impact of such an interplay on varying membrane disruption mechanisms using sequence alterations in an archetypal HDP. Such a peptide would span >3 nm in a folded state, comprise specialist residues or motifs with an apparent separation into sub-domains and exhibit a preferential membrane-disruption mechanism. We have identified Cecropin B (CecB) as an ideal candidate to meet the above requirements and test our hypothesis by considering carefully chosen modifications of the peptide as shown in Fig. 1 and discussed in more detail below.

## Results and Discussion

CecB, originally isolated from the cecropia moth *Hyalophora cecropia*, belongs to a super-family of α-helical HDPs^36^. It comprises 35 amino-acid residues that arrange into classical heptad repeats allowing for the formation of an amphipathic helix spanning ~5.25 nm (0.54 nm per turn)^37^. The first (*i*) and the last (*i* + 7) residues in each heptad tend to be of the same type (i.e. polar or hydrophobic) (Fig. 1a and Table S1). In the folded helix, the residues are adjacent when viewed along its main helix, thus allowing the residues to segregate into opposite polar and hydrophobic faces. Resulting amphipathic helices orient more parallel to the surface of anionic microbial membranes disrupting their phospholipid bilayers via a detergent-like carpet mechanism (Fig. 1c)^27,38^. CecB has three abutting and overlapping G(X)_n_G motifs that stretch from the second heptad of the N-terminus. This suggests that the first 12 residues are primarily responsible for the helix formation and antimicrobial activity, which is also consistent with the importance of the N-terminal tryptophan (W2) for the activity (Fig. 1a,b)^27^. The contiguous stretch of the three glycine zippers subdivides the sequence into three sub-domains of comparable lengths (Fig. 1a). This structure compromises autonomous helix formation in solution and helps the helix to adapt to membrane curvature^8^. As the peptide reaches a threshold concentration membrane curvature increases, but without necessarily leading to poration^16^. The primary role of insertion is therefore to stretch, disorder and thin the outer leaflet of the bilayer^38^, which proves sufficient for detergent-like mechanisms^15^. These assume a surface-bound state for peptide helices without a subsequent transmembrane insertion^18^. Instead, carpet-forming peptides intercalate below the glycerol backbone of the head groups forcing the expansion and thinning of the outer leaflet^8,39^. Poration for these peptides, as a result of transmembrane insertion, is deemed unnecessary and difficult to accommodate. For instance, the length of CecB helices (5 nm) exceeds the thickness of the bilayer (<4 nm) imposing an implicit bias towards a non-poration mechanism (Fig. 1c)^38^.

### Visualising mechanistic disruption in reconstituted phospholipid membranes

With these mechanistic models in mind, we monitored the impact of CecB on reconstituted phospholipid membranes using lipid compositions that produce fluid-phase bilayers at biologically relevant temperatures^21,40^. Anionic unilamellar vesicles (AUVs) and zwitterionic unilamellar vesicles (ZUVs) provided microbial and mammalian membrane mimetics, respectively. 1,2-dilauroyl-sn-glycero-3-phosphocholine (DLPC) was used to assemble ZUVs, whereas its 3:1 molar mixture with 1,2-dilauroyl-sn-glycero-3-phospho-(1′-rac-glycerol) (DLPG) was used to assemble AUVs^20,25^. As gauged by Circular dichroism (CD) spectroscopy, in AUVs CecB folded into a strong α-helix at micromolar chain concentrations, whereas no appreciable structure was found in aqueous buffers and ZUVs (Fig. S1a).

**Figure 2:**
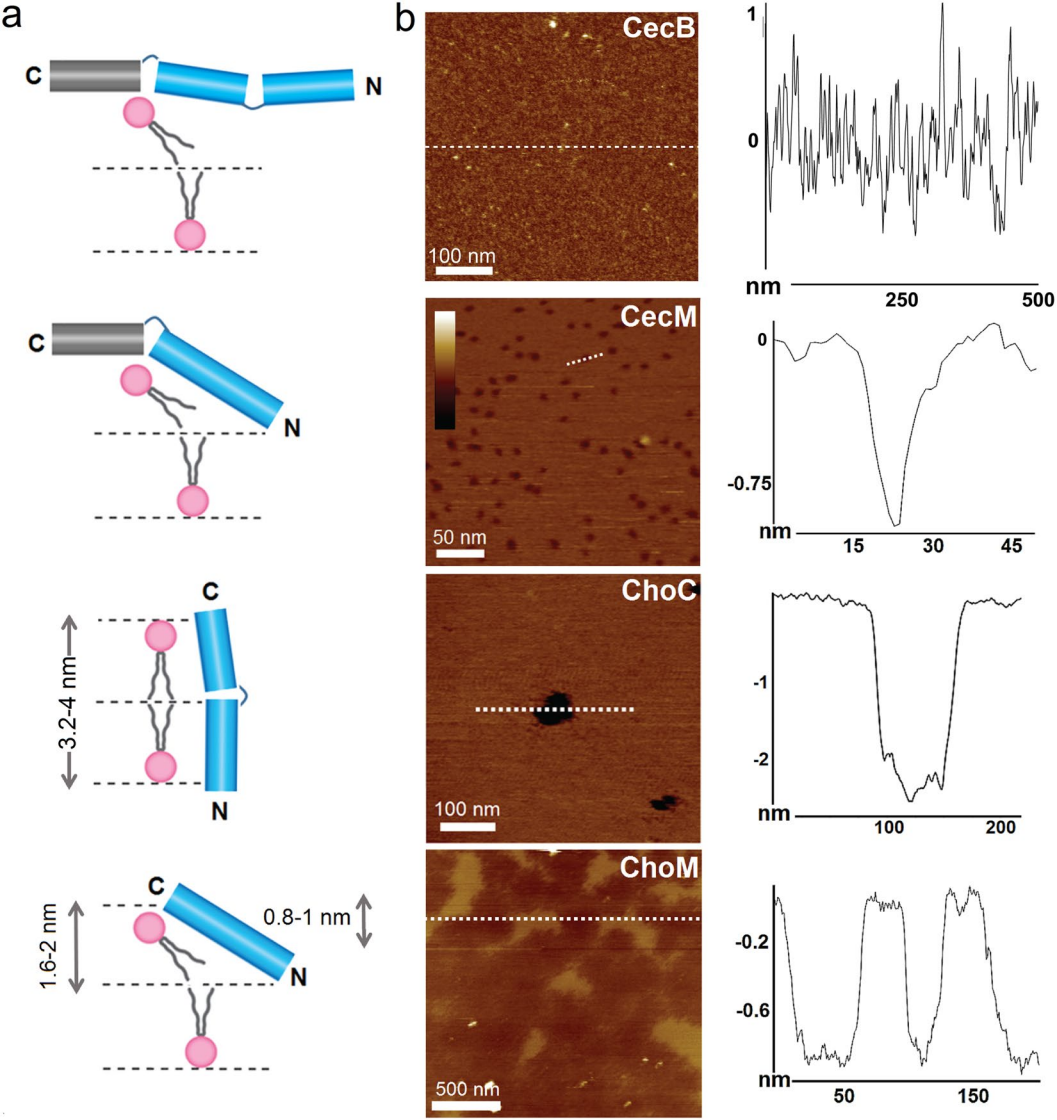
Membrane disruption mechanisms in DLPC/DLPG (3:1, molar ratio) supported lipid bilayers. (**a**) Schematic representations of relative orientations of cecropin peptides in lipid bilayers: (from top down) CecB, CecM, Choc and ChoM. Designations are as in Fig. 1. (**b**) Topography of SLBs treated with cecropin peptides (left) and cross-sections along the highlighted lines (right). The images were taken at 90 min (CecB), 15 min (CecM), 10 min (ChoC) and 6 min (ChoM) of incubation. Color scale is 6 nm.

To visualize directly the impact of CecB folding on the membranes, supported lipid bilayers (SLBs) were prepared by the surface deposition of the AUVs on appropriate substrates as previously described^21^. Such preparations are homogeneously flat, to within ~0.1 nm in their unperturbed state, and offer ideal substrates for accurate depth measurements by atomic force microscopy (AFM) in aqueous solution and in real time (Fig. S2)^25,40^. AFM analyses of SLBs incubated with CecB revealed corrugated surfaces without apparent poration over 90 min, suggesting that CecB denatures the bilayer in a non-cooperative manner (Figs 2 and S3)^38^. This mechanism is consistent with the primary structure of the peptide (Figs 1 and 2a).

Firstly, the C-terminal domain of CecB is preceded by a well-defined AGPA motif, the so-called hinge moiety, which bends the helix at an obtuse angle inducing significant flexibility in the region^41^. This enables the domain to fold independently of the N-terminal domains and act as an adaptable anchor lying flat on the membrane surface^15,18^. The domain incorporates small alanyl residues and is net neutral (Fig. 1a), which ensures weaker membrane binding. This is in stark contrast to hydrophobic and cationic residues, which have strong affinity to anionic membranes and appear exclusively in the N-terminal domains (Fig. 1a).

Secondly, the two N-terminal domains are continuously cationic, while the second (middle) domain features arginine residues straight after the first glycine residue (Fig. 1a). Arginines provide tighter and more extensive electrostatic interactions with anionic phospholipids when compared to lysines^42^. Therefore, their incorporation into the first glycine zipper, which generally weakens helicity, is to partially recover it by strengthening electrostatic interactions. Such helix-tuning supports membrane-mediated folding.

Thirdly, the peptide is believed to form small channels at concentrations that are lower than minimum inhibitory concentrations (MICs)^43^. Though such channels have yet to be observed, we reason that CecB has an intrinsic propensity for pore formation. We further reason that this propensity can be enhanced by (i) discarding the first and the last glycyl residues in the sequence, thus retaining only the middle glycine zipper, and (ii) reshaping the *i*, *i* + 7 heptad pattern into *i*, *i* + 3 and *i*, *i* + 4 spacing pattern^21^. The latter is common for α-helical coiled coils and arranges cationic residues in a continuous seam exposed to oppositely charged moieties and hydrophobic residues in another seam promoting hydrophobic interfaces^44^. As a result, cooperative inter-helical interactions are enabled in anionic amphipathic environments, such as anionic membranes, leading to locally oligomerized helices. The resulting sequence is a two-domain structure, termed cecropin mutant or CecM (Fig. 1a). The peptide has one uninterrupted N-terminal domain whose length matches the bilayer thickness, allowing it to fully insert into the bilayer. Yet, the remaining glycine zipper in conjunction with the C-terminal domain hinged on the membrane surface by the AGPA motif can be expected to arrest transmembrane poration or prevent it altogether (Fig. 2a).

### Converting carpet-like disruption into poration

**Figure 3:**
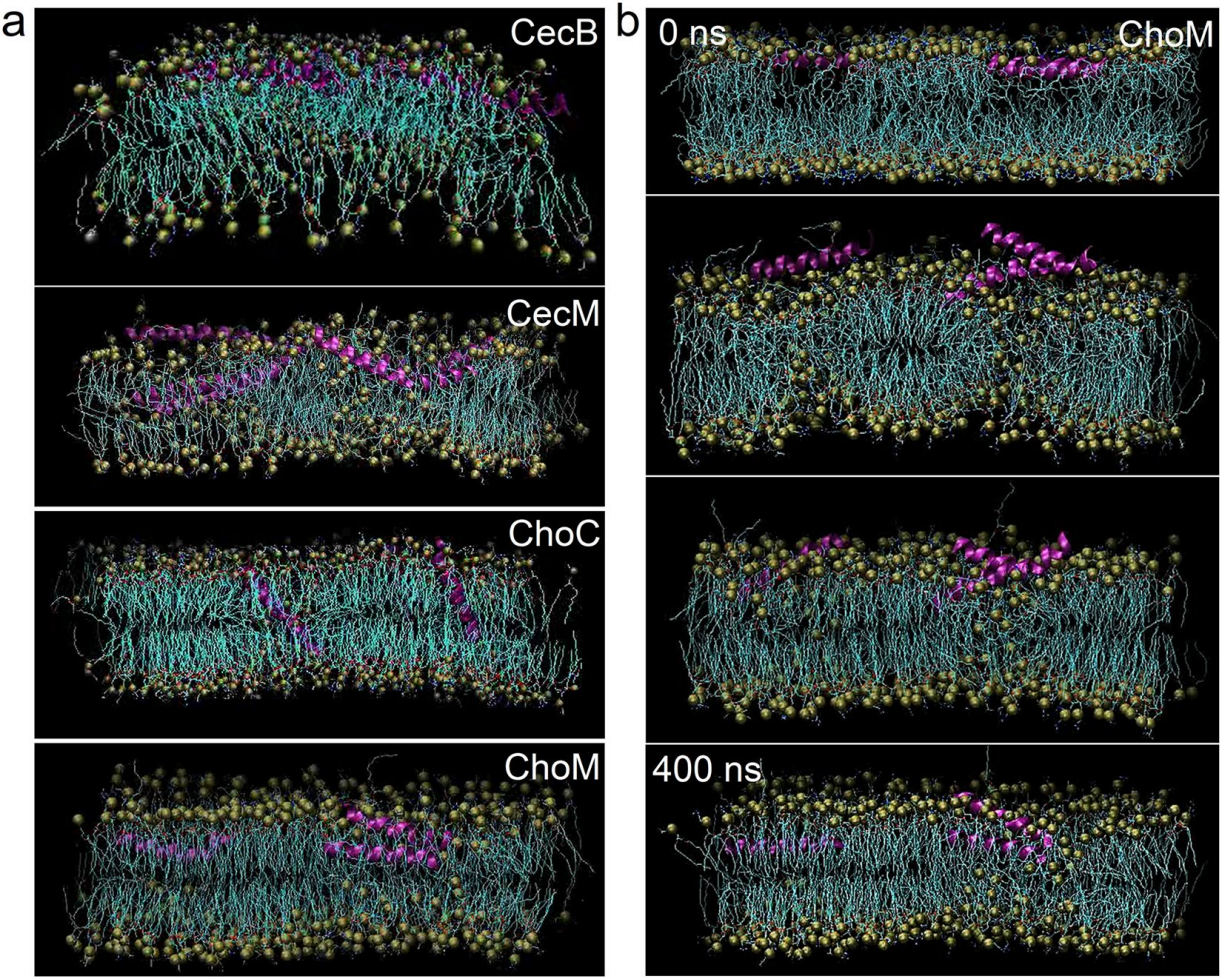
Peptide orientations in membranes. (**a**) 0.5 µs snapshots of molecular dynamics simulations for individual cecropin peptides in AUVs. (**b**) A representative simulation for ChoM following an equilibration phase (0 ns). Key: peptide helices are in magenda, lipid aliphatic chains are in cyan, phospholipid headgroups are green spheres (see also Movies S1–S4).

Consistent with these conventions, CecM folded in AUVs, but not in aqueous buffers or ZUVs (Fig. S1b). As expected for helical assemblies and coiled-coils, spectral Δε_222_/Δε_208_ ratios were ≥1^45^. In comparison, the ratios of <1 for CecB suggest that monomeric helical conformers were predominant for the parent peptide (Fig. S1a). Unlike CecB, CecM formed abundant pores of 9 ± 2 nm diameter and ~1.5 nm in depth over the first 10 min of incubation in SLBs (Figs 2b and S3). A priori, these observations and in particular the observed pore depths are consistent with monolayer poration^25^. However, given the small size and (V) shape of the pores, it cannot be excluded that the AFM tips used to measure the pores were not sharp enough to probe the full penetration depth in these experiments. Notwithstanding, the pores appeared remarkably homogeneous suggesting that poration is arrested laterally and possibly vertically. To gain a better insight into this process, the dynamics and modes of insertion for both peptides were assessed using molecular dynamics (MD) simulations. Different initial configurations with respect to the membrane surface were used including flat, transmembrane or tilted (Fig. S4). Regardless of the configurations, the behaviours of the two peptides were drastically different. Over the half microsecond time scales, CecB formed rod-like helices that oriented parallel to the membrane surface. The helices intercalated below the head groups of the outer leaflet, which largely explains the preference for carpet-like mechanisms (Fig. 3a and Movie S1). CecM helices, on the other hand, rapidly adopted a kinked conformation that assumed tilted orientations with the N-terminus of the peptide sinking deep into the bilayer interface (Fig. 3a and Movie S2). Simulations showed a greater helical content for CecM when compared to CecB (Table S2 and Fig. S5). At no point during the simulation did the peptide adopt a transmembrane orientation (Movies S1 and S2). Instead, it tended to cluster phospholipids into oligomeric structures, in accord with observations by others for other HDPs^14,46^. Combined with the experimental evidence, the MD results indicate the preference of CecM to form fixed pores of relatively small sizes as a characteristic signature.

The findings also re-emphasise the role of the C-domain as a topological constraint of poration, which may regulate pore sizes and penetration depths. Therefore, truncating CecB at the start of the AGPA motif was expected to remove such a constraint prompting more aggressive and heterogeneous poration. Indeed, the N-terminal part of CecB, dubbed chopped cecropin (ChoC), formed larger pores of varied sizes, allowing to confidently measure the pore depths of ~2.7 nm, which conformed to ChoC spanning the hydrophobic core of the bilayer (Figs 2 and S3). The full length of the peptide (~3.2 nm) matches the thickness of the bilayer, allowing for the complete insertion of the peptide in the membrane (Fig. 2a). In addition, MD simulations confirmed that ChoC helices immediately adopted a transmembrane configuration, which remained stable over the half microsecond simulation following an equilibration phase (Fig. 3a and Movie S3). ChoC exhibited appreciable helicity in AUVs, with spectral characteristics (Δε_222_/Δε_208_ < 1) typical of monomeric helices (Fig. S1c). As outlined above, the arginyl residues within the retained glycine zipper may regain or strengthen helix formation, which also suggests that breaking helicity is not the main function of the zipper. More likely, the motif is meant to discriminate against inter-helical associations in favor of monomeric helices, given it takes at least three abutting heptads to interdigitate an α-helix^47^. ChoC matches the length, but falls short of three contiguous heptads because of the zipper. The same holds true for CecB, whereas the N-terminal domain of CecM stretches nearly three heptads to the only interruption by the first glycyl residue (Fig. 1a). Note should be taken however that only high resolution structural elucidations may reveal the exact nature of the conformational changes observed in the sequence mutants.

### Non-porating membrane exfoliation

A modified sequence, dubbed ChoM, was produced to replace the two glycines with isoleucyl and lysyl residues, giving rise to an uninterrupted helix with a similar polar angle (Fig. 1b). CD spectra confirmed the responsive folding of the peptide with an apparent tendency for inter-helical associations in AUVs (Δε_222_/Δε_208_ > 1), resembling that of CecM (Fig. S1b,d). Unlike CecM, ChoM is free of a-priori constraints for membrane binding and insertion and lacks an apparent coiled-coil pattern that could otherwise aid in restricting oligomerisation in the membrane^48^.

As a consequence, ChoM cannot arrest pore formation and is promiscuous in lipid binding (Fig. 1a). This is further compounded by that arginines are no longer confined within a helix breaker and can form cooperative electrostatic networks on the membrane surfaces, which may endow the peptide with substantial freedom of lateral movement without the need to insert and orient in the membranes^25^. Indeed, MD simulations showed cooperative interactions between arginines and anionic phospholipids for ChoM, which were greater in numbers when compared to the other three peptides (Fig. S6). As judged by AFM, the peptide did not assemble into pores or corrugated SLBs as CecB does. Instead, it exfoliated the bilayers within the first ten minutes of incubation (Figs 2b and S3). The measured depth profiles were consistent with the progressive removal of the outer leaflet of the bilayer. Comparable results of monolayer poration were obtained for thicker SLBs (~4 nm), which were assembled as 3:1 molar mixtures of 1-palmitoyl-2-oleoyl-sn-glycero-3-phosphocholine (POPC) and 1-hexadecanoyl-2-(9Z-octadecenoyl)-sn-glycero-3-phospho-(1′-rac-glycerol) (POPG) lipids (Fig. S7). The effect was distinct from that of CecB which corrugated the SLBs in the same manner observed for the DL-type membranes (Figs 2, S3 and S7). These thicker bilayers contain unsaturated lipids, which alone cannot support an orientational order and their thicknesses exceed the span of the folded ChoM. The saturated lipids of DLPC/DLPG bilayers are more densely packed and their thickness (3.2 nm) can match the complete transmembrane span of the folded ChoM. However, irrespective of these differences, ChoM promoted the same poration mechanism in both membrane types. The observed effect may also suggest that more precise variations in poration in different lipid types may not be fully ascertained in supported lipid bilayers. The results are yet consistent with that HDPs are universal membrane disrupters that lyse microbial cells, not necessarily specifically, but rapidly within the time limits of their proteolytic stability^6–9,18^. Complementary to folding characteristics in DL-type bilayers, CD spectra for CecM and ChoM in the thicker membranes were distinctive from those for ChoC and CecB (Fig. S8).

The exfoliation process was reminiscent of phenomena observed in outer leaflets exposed to prion proteins or bacterial cassette transporters^49,50^, although in these cases monolayer pits, as opposed to monolayer exfoliation, occur. Another analogy can be found in monolayer stalks formed by amphiphysin N-BAR domains during membrane fusion^51^. Of more direct relevance, however, is a mechanistic model, which proposes that HDPs act by splaying phospholipids of the outer leaflet^8^. This mode of action does not make a particular reference to poration and places a stronger emphasis on the thinning of bacterial membranes as a means of membrane rupture. It also assumes an oblique orientation of peptide helices with respect to the membrane surface. Previously, we have shown that such an orientation can lead to rapid antimicrobial mechanisms targeting the outer leaflet of the bilayer^25^. However, membrane exfoliation ensued via monolayer poration, which was not observed for ChoM. Non-poration exfoliation is intriguing because it does not fall under either category of pore or carpet formation. According to MD simulations, ChoM has a clear preference to intercalate under the phospholipid headgroups of the outer layer without attempting to span the membranes (Fig. 3a and Movie S4). Similar to CecM the peptide tended to arrange into higher oligomers in contrast to ChoC and CecB, which showed preference for monomeric and dimeric forms (Fig. S9). The simulations also revealed that the ChoM helices were only marginally tilted with respect to the membrane surface (Fig. 3b), and that on average they appeared as more rigid and less curved than ChoC, thus introducing local conformational changes fostering interactions of arginine residues in membranes (Fig. S10).

**Figure 4:**
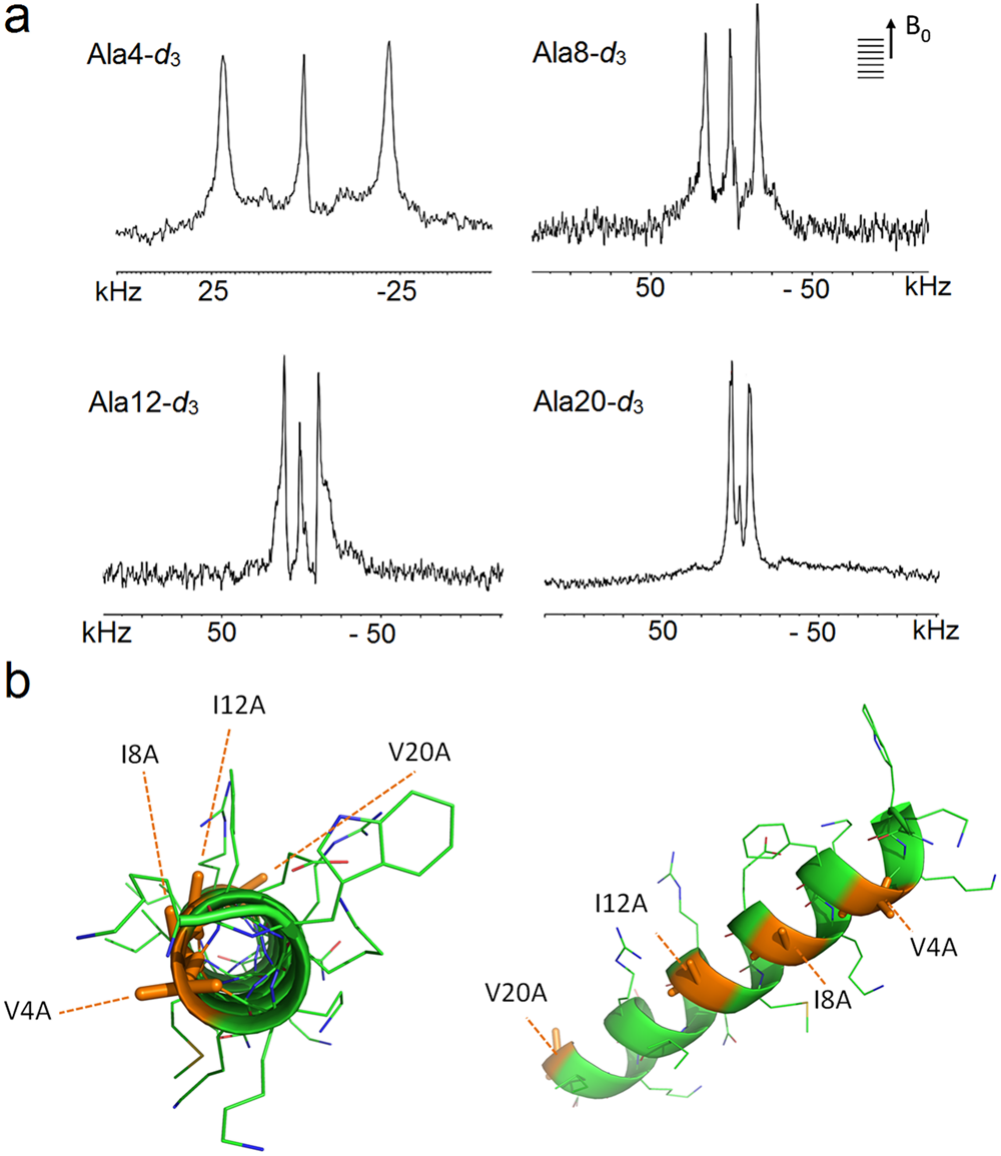
ChoM orientation in AUVs. (**a**) Oriented ^2^H NMR quadrupolar splittings in AUVs (POPC/POPG, 3:1 molar ratio) at L/P ratio 25 for four ChoM mutants each with a single deuterated alanine (Ala-*d*_3_) mutation at positions 4, 8, 12 and 20, respectively. (**b**) A GALA-derived helix model showing relative positions of labelled Ala-*d*_3_ (brown).

Together with the rapid exfoliation observed by AFM, these results imply that ChoM helices are held in the headgroup layer by strong electrostatic interactions causing a large degree of disruption. Indeed, as demonstrated by solid state ^2^H NMR (ssNMR) spectra obtained in AUVs assembled from phospholipids with deuterated acyl chains, there was significant disorder in both lipid components, suggesting an in-planar peptide orientation (Fig. S11)^52^. At decreasing lipid-to-peptide (L/P) ratios (increasing peptide), ChoM induced clear segmental ordering in lipids, with reduced ^2^H quadrupolar splittings indicating a more pronounced membrane disruption upon binding more peptide (Fig. S11, Table S3). A larger disrupting effect is observed for the anionic lipid component suggesting a charge-driven interaction. The results are consistent with the peptide intercalating in the interface region of the bilayer and suggest a synergistic and charge-driven mode of action causing membrane thinning, estimated from the observed order changes to be 3.6 Å and 5.5 Å for the neutral and anionic components, respectively^52^. To complement the data, angular constraints informing the orientation of the peptide with respect to the bilayer normal were obtained using the geometric analysis of labelled alanines (GALA) by oriented ssNMR spectroscopy (Figs 4, S12). Four ChoM peptides, each being the ChoM sequence mutated at a single position with a deuterated alanine (Ala-*d*_3_) (Table S4). All selectively deuterated residues were resolved in large quadrupolar splittings indicating a non-transmembrane orientation (Figs 4a, S12). A minor second component was apparent for the C-terminal half of the peptide, which is suggestive of more heterogeneous orientation or partial fraying of ChoM helices in the region (Table S5, the distinct populations are interchanging slowly: >10^−6^ s). At lower L/P ratios (increased peptide concentration) an increased distortion of lipid bilayers was expected as a larger proportion of lipids would interact with peptide (Fig. S12). Since bilayer disordering is typically more pronounced for peptides splaying the upper leaflet than for transmembrane peptides^52^, the effect could be attributed to that the peptide remained in the bilayer in its designed anchored position.

The fitting of the individual deuterium constraints to root-mean-square deviation plots returned an overall orientation for all of the four mutants as nearly parallel to the membrane surface, within a marginal tilt of 2° +/− 8° for the ensemble (based on a maximum RMSD of 10 kHz for an error estimate, Fig. S13). Based on these results, it can be concluded that ChoM intercalates and assembles close to the hydrophobic interface in the bilayer brushing through the interface and irreversibly perturbing the bilayer with membrane exfoliation as a result. In terms of biological activity, we hypothesize that this mechanism should favour a faster attack on bacteria, manifesting in faster killing rates when compared to those for the other three cecropin peptides as described below.

### Comparative biological activity

Consistent with the above results and discussion, live/dead assays revealed a nearly complete lysis of *E*. *coli* and *B*. *subtilis* cells by the first 15 min of exposure to ChoM, with no cells surviving within the first cell doubling (20 min) (Fig. S14). The data closely correlated with the exfoliation rates observed by AFM. Similarly, ChoC that exhibited pore formation developing over an hour in reconstituted membranes tended to reach quantitative killing rates just after the first hour of incubations. CecM proved to have comparable rates with those of ChoC suggesting that the differences in pore sizes and morphology caused by the two peptides have little or no effect on killing kinetics (Fig. S15). The analysis of membrane disruption by CecB was complicated by the absence of clearly recognisable features in the disrupted reconstituted membranes (Figs 2 and S3). This hampered a mechanistic interpretation of its killing kinetics. Nevertheless, the peptide showed comparable killing rates with those for CecM and ChoC (Figs S14 and S15).

In native hosts, HDPs are secreted to tackle opportunistic pathogens within minutes, their proteolytic life time. Therefore, the observed rates, both in reconstituted and live membranes, indicate that the disruption mechanisms of this cecropin series become apparent within the timescale of native biological responses. The live-dead assays provide accurate estimations of antimicrobial action at the level of individual cells. However, the tests are devoid of contributions from other factors such as the phenotypic tolerance of bacteria to antibiotics and inoculum effects^32^. Optical density measurements performed for the bulk of bacterial culture over much longer periods of time give more generic estimations of biological activity. Expressed as MICs the results of these measurements refer to the lowest concentrations of tested agents at which bacteria show no visible growth. All four peptides were found to have MICs in the low range that is typical of potent antimicrobial agents (Table 1). CecB showed distinctively lower activities against *B*. *subtilis*, which is somewhat surprising given that killing kinetics of the peptide were similar to those of ChoC and CecM in the first hour of treatment (Fig. S14). This indicates that the peptide failed to affect bacterial growth over longer incubations, suggesting that such incubations may be more profoundly subject to inoculum effects or peptide depletion during the process. Similarly, all GALA peptides, which constitute a short alanine scan of the sequence, maintained antimicrobial activities at comparable levels, with the exception of *S*. *aureus* (Table S6). The GALA peptides were less active against the bacterium. Partly, this can be explained by that the alanine scan reduced the overall hydrophobicity of each peptide, having also an effect on binding affinities of peptide molecules to membranes. However, with no significant variations in activity against the other bacteria tested, the exact nature of this effect is unclear. It may be attributed to that local conformational changes in GALA peptides may be more prone to the partial folding and pre-oligomerisation of the peptides in thicker peptidoglycans on the surface of *S*. *aureus* leading to the inhibition of peptide migration to the cytoplasmic membrane^53^.

**Table 1:** Biological activities of antibiotics and peptides used in the study.

cell	Compound						
	daptomycin	tetracycline	ceftriaxone	CecB	CecM	ChoC	ChoM
	*Minimum inhibitory concentrations*, *µM*						
*P*. *aeruginosa*	>100	<6	>25	<1.5	<1.5	<1.5	<1.5
*S*. *aureus*	<7	<0.5	<3	>100	>25	>100	<3
*E*. *coli*	>100	<1.5	<6	<1	<1.5	<6	<3
*B*. *subtilis*	<7	<0.5	<1	>50	<7	<5	<1.5
*S*. *typhimurium*	>60	<1	<1	<3	<1	>12	<3
*M*. *luteus*	<15	<0.5	<0.5	<1	<1	<1	<0.5
	(*LC*_50_)^a^, *µM*						
*HE* ^b^	>250^c^	UD	UD	>250^d^	>250^d^	>250^d^	>250^d^

^a^Median (50%) lethal concentration. ^b^Human erythrocytes. ^c^10% hemolysis. ^d^<1% hemolysis. UD is for undetectable (>1000).

CecB and ChoC were also virtually inactive against *S*. *aureus*, though lower activities against Gram positive strains did not appear systematic. By comparison, daptomycin – a membrane-active antibiotic that preferentially porates Gram positive membranes – selectively inhibited Gram positive bacteria. Intracellular antibiotics, tetracycline and ceftriaxone, were equally effective against all bacteria tested, demonstrating expected broad spectrum activities. Amongst the four cecropin peptides, only ChoM gave notably low MICs against all strains used (Table 1), suggesting a distinctive antimicrobial mechanism.

Non-differential antimicrobial responses reflect the nature of HDPs as non-specific agents that rapidly kill pathogens on contact. In many cases this carries an additional cost of hemolytic effects given the weakly anionic surfaces of erythrocytes^32^. In the studied series, except daptomycin that was weakly hemolytic, hemolysis was not apparent (Table 1 and S6). Daptomycin is a net negative molecule, but decisively hydrophobic, which makes it more related to host defense toxins exhibiting extensive hydrophobic faces. However, it does not target Gram negative bacteria, against which the cecropin peptides were potent. HDPs bind to lipid bilayers as monomers, which then assemble into pores or carpet-like structures. These form continuously and lead to the progressive disruption of lipid bilayers. Such mechanisms have been observed in both lipid bilayers and live bacteria^21,25^, and the timescales of these physical events are consistent with the rapid lysis of bacterial cells. Complementary to non-specific binding to bacterial membrane bilayers, the peptides can also bind to lipopolysaccharides (LPS) of Gram negative cell walls and their precursors, which have been proposed as primary targets for HDPs^54^. Variations in the activities against Gram negative strains did take place in the cecropin series, with ChoC being noticeably weaker against a Gram negative *S*. *typhimurium*.

### Probing bioactivity variations in cross-resistant bacteria

To explore these variations, the peptides were tested against cross-resistant strains of *S*. *typhimurium*, which were experimentally derived from the wild type bacterium subjected to increasing concentrations of pore-forming human HDPs^55^. Three genetic mutations were selected as candidate contributors to resistance and were reconstituted in the wild type genetic background, giving rise to the strains given in Table 2. One strain has the *waaY* gene mutated. This gene encodes for WaaY kinase that adds a phosphate group to the heptose II residue in the LPS inner core. The deactivation of the gene leads to decreased susceptibility to HDPs^56^, as a result of reducing peptide binding to cell surfaces to the levels below those necessary to lyse the bacterium^57^. Two other strains were mutated in *pmrB* and *phoP* genes, both of which are involved in masking phosphate groups (diminished charge) and influencing membrane fluidity (restricted motion)^58^. Finally, the fourth strain combined all three mutations (Table 2).

**Table 2:** Cross resistance of *S*. *typhimurium* to antimicrobial agents used in the study.

Compound	Mutant^a^				
	DA6192	DA22427 *waaY* (del bp17FS)	DA23175 *pmrB* (R13H)	DA23307 phoP (D23N)	DA23899 *waaY*, *pmrB*, *phoP*
	*Minimum inhibitory concentrations*, *µM*				
polymyxin B	<0.5	<0.5	<0.5	<2	<6
amhelin^20^	>6	>25	>12	>12	>50
tilamin^24^	>3	>6	>25	>12	>50
CecB	<3	<3	<3	<3	>6
CecM	<1	<1	<1	<2	<2
ChoC	>12	>12	>25	>25	>25
ChoM	<3	<6	<6	<6	<6
tetracycline	<1	<1	<1	<1	<1
ceftriaxone	<1	<1	<1	<1	<1

^a^Original nomenclature is used^55^.

Antimicrobial activities measured against these strains revealed two major trends. Corresponding MICs for the cecropin peptides were doubled when compared to those against the wild type bacterium. Although the strains appeared as more resistant, overall the MICs remained low, with the exception of ChoC, for which the two-fold decrease essentially meant the loss of activity (Table 2). Although this could be for a variety of reasons, this effect did not seem to relate to the *waaY* mutation, but did to the other two mutations, albeit without a dominant impact from one or another. Because ChoC was the only peptide in the series that formed transmembrane pores, one may expect that poration could be more susceptible to such basic alterations in membranes. Indeed, the two pore-forming synthetic peptides that were as active against the wild type of *S*. *typhimurium* as were the cecropin peptides, were found inactive against the strain combining all three mutations (Table 2). A similar trend of weakened activity was observed for polymyxin B – an endotoxin-binding antibiotic which depolarises Gram negative membranes by forming ion-permeable pores. However, transmembrane poration by the antibiotic does not seem to be a primary cause of bacterial death^59^. Polymyxin B is known to aggregate with LPS on the surfaces of *S*. *typhimurium* cells forming the so-called blebs in the outer leaflet of the bacterium membrane^60^. The process is not exactly associated with membrane exfoliation or monolayer poration, but does require acidic phospholipids to increase membrane permeability^61^. ChoM, which does not form visible pores but disrupts the outer leaflet of bacterial membranes, shared the same level of activity against the resistant strain. In this light, CecM, which formed pores and gave the lowest MICs against all four strains, may only disrupt outer leaflets with small pits that are nonetheless sufficient to kill the bacteria. The results also confirm that the activity of membrane-active antibiotics and HDPs depends on the efficiency with which they target bacterial membranes. Synergistic and more profound effects by HDPs concern the relative distributions of charged and hydrophobic residues and their interaction interplays in bilayer interfaces, which is in clear contrast to intracellular antibiotics that use different pathways to cross bacterial membranes. Indeed, the acquired cross-resistance of *S*. *typhimurium* proved to be ineffective against tetracycline and ceftriaxone (Table 2).

## Conclusion

The original CecB adopts a helix of imperfect amphipathicity with polar residues occupying the hydrophobic face and vice versa (Fig. 1a)^14^. The polar residues promote membrane binding, but also interfere with the insertion of hydrophobic residues into membranes and consequently inhibit interfacial structuring deep inside the bilayer^25^. Imperfect amphipathicity can be balanced by a neutral alanyl cluster separating two asymmetric cationic facets^21^. In ChoC the glycine zipper partitions hydrophobic and polar residues by embedding the hydrophobic face, which breaks helix cooperativity exactly at the polar-hydrophobic interface (Fig. 2a). This may be gradually stabilized in membranes, arranging nearly perfect amphipathicity characteristic of transmembrane helices^21^. Replacing the glycine zipper in ChoC with single hydrophobic and cationic residues result in a much faster migration dynamics, which monolayer poration can readily facilitate (Fig. 2)^25^. Equally, introducing multiple cationic and hydrophobic faces in CecB can enhance imperfect amphipathicity by allowing contiguous electrostatic and hydrophobic pairings that would support interfacial contacts in lipid bilayers as well as tight packing of monomeric helices into low-oligomer bundles (Fig. 1a)^27^. The bundles introduce three-dimensional equipotential surfaces with limited freedom for translational and rotational motion thereby settling for a restricted mode of poration^46^. As anticipated, CecM assembled into compact, small and shallow pores agreeing with a conserved mechanism engaged in the outer leaflet of the bilayer (Figs 2b and S3). ChoM instead exhibited persistent intercalation dynamics accompanied by the cooperative and fast removal of monolayer phospholipids in minutes (Figs 2b and S3). Given that all four of these peptides were strongly antimicrobial and non-hemolytic (Table 1) and that their biological activities correlated with their preferential folding in AUVs, and not in ZUVs (Fig. S1), the study permits a collective conclusion that HDP sequences allow for the tuneable disruption of microbial phospholipid bilayers.

Emerging bacterial strains also develop different molecular strategies: to alter cell surfaces and endow them with cross-resistance toward membrane-active antibiotics. Now classical approaches include Gram-positive methods of neutralising cell walls by modifying teichoic acids with D-alanine^61^ and more recently discovered tools of Gram-negative pathogens to fortify LPS with phosphoethanolamine conjugated to phosphate groups^62^ or via the glycylation of aliphatic acyl chains^63^. All such modifications, however subtle, confer resistance to HDPs. Conversely, antimicrobial sequences themselves present cryptic, but practical, strategies for circumventing evolving resistance mechanisms^64^. As it was long proposed^7^, developing a widespread resistance against HDPs, which are evolutionarily conserved molecules, would carry a high cost. Although resistance mechanisms against HDPs do emerge, these are not universally applied across even one family and are readily counteracted by seemingly marginal alternations in peptide structure. As demonstrated here, it is the intrinsic property of an HDP to express amenable membrane-disruption mechanisms, which can be tuned using simple mutations. We find this property remarkable as it indicated and exploits a phenomenal structural plasticity of the peptides with respect to their function: irrespective of their exact origin, sequences and modes of action, HDPs continue to target microbial membranes in one way or another. A critical question that never ceases to amaze is why both natural and engineered HDPs occur in such numbers and diversity.

To a large extent, this could be explained by that HDPs exhibit multiple mechanisms of action including intracellular targeting and binding to cell-surface receptors. Similarly, microbial resistance mechanisms are not limited to cell surface or membrane modifications and may involve efflux pumps that block the passage of HDPs into the cell or the secretion of peptide effectors that block access to cell membranes^65,66^. Therefore, the host-pathogen arms race is also likely to involve multiple and simultaneous adaptions in bacteria that may be matched or counteracted by those in evolving HDPs.

This work provides at least a partial and complementary answer. Namely, each archetypal HDP remains a well optimised structural template that combines a repertoire of different mechanisms, the best of which can be selected to execute effective antimicrobial responses. Consequently, these designs can offer the discovery of distinct antimicrobial mechanisms that can benchmark inter-relationships between poration and carpet-like mechanisms encoded by a given HDP.

## Methods

### Peptide Synthesis and Purification

All peptides were assembled on a Liberty-1 microwave peptide synthesizer (CEM Corp.) as peptide amides using solid phase Fmoc/tBu protocols, HBTU/DIPEA as coupling reagents and Rink amide 4-methylbenzhydrylamine resin (Novabiochem, UK). Fmoc-Ala-OH-3, 3, 3-d_3_ (Sigma Aldrich) was used for the synthesis of GALA d_3_-peptides. Following post-synthesis work-up and purification the identities of the peptides were confirmed by analytical RP-HPLC and MALDI-ToF mass spectrometry. MS [M + H]^+^: CecB, *m/z* 3834.6 (calc), 3836.0 (found); CecM, *m/z* 3968.1 (calc), 3969.1 (found); ChoC, *m/z* 2572.2 (calc), 2573.5 (found); ChoM, *m/z* 2699.4 (calc), 2703.3 (found); V4Ad_3_-ChoM, *m/z* 2674.4 (calc), 2675.4 (found); I8Ad_3_-ChoM, *m/z* 2660.3 (calc), 2664.5 (found); I12Ad_3_-ChoM, *m/z* 2660.3 (calc), 2665.1 (found); V20Ad_3_-ChoM, *m/z* 2674.4 (calc), 2678.1 (found).

### High Performance Liquid Chromatography

Analytical and semi-preparative gradient RP-HPLC was performed on a JASCO HPLC system using Vydac C18 analytical (5 μm) and semi-preparative (5 μm) columns. Both analytical and semi-preparative runs used a 10− 60% B gradient over 50 min at 1 mL/min and 4.7 mL/min respectively with detection at 230 and 220 nm. Buffer A – 5% (vol/vol) and buffer B – 95% (vol/vol) aqueous CH_3_CN, 0.1% TFA.

### Minimum Inhibitory Concentration Assay

Minimum inhibitory concentrations (MICs) were determined by broth microdilution on P. aeruginosa (ATCC27853), E. coli (K12), S. aureus (ATCC6538), M. luteus (NCIMB13267), B. subtilis (ATCC6633), S. typhimurium (DA6192) and E. faecalis (OG1X) according to the Clinical and Laboratory Standards Institute and as recommended for antimicrobial peptide testing^67^. Typically, 100 μL of 0.5–1 × 106 CFU per ml of each bacterium in Mueller Hinton media broth (Oxoid) were incubated in 96-well polystyrene microtiter plates with 100 μL of serial twofold dilutions of the peptides (from 100 to 0 μM) at 37 °C on a 3D orbital shaker. The absorbance was measured after peptide addition at 600 nm using a Victor 2 plate reader (Perkin-Elmer). MICs were defined as the lowest peptide concentration after 24 h at 37 °C. All tests were done in triplicate and results are summarised in Table 1. The mutant strains of S. typhimurium, named using the original nomenclature, were kindly provided by Dan Andersson^55^. All tests for the mutants were done in triplicate and results are summarised in Table 2.

### Hemolysis Assay

Hemolysis was determined by incubating a 10% (vol/vol) suspension of human erythrocytes with peptides. Erythrocytes were rinsed four times in 10 mM phosphate buffer saline (PBS), pH 7.2, by repeated centrifugation and re-suspension (3 min at 3000 × g). Erythrocytes were incubated at room temperature for 1 h in either deionized water (fully haemolysed control), PBS, or with peptide in PBS. After centrifugation at 10,000 × g for 5 min, the supernatant was separated from the pellet, and the absorbance was measured at 550 nm. Absorbance of the suspension treated with deionized water defined complete hemolysis. The values given in Table 1 correspond to concentrations needed to kill a half of the sample population (50% lysis of human erythrocytes) and are expressed as median lethal concentrations—LC_50_. All tests were done in triplicate.

### Stain-Dead Antimicrobial Assay

*E*. *coli* and *B*. *subtilis* cells were centrifuged to give a cell pellet, which was washed twice with 10 mM phosphate buffer (pH 7.4) before being reconstituted in phosphate buffer. 100 µl of the solution was dispensed in an 8-well chamber (LabTek) with diluted (1/500) propidium iodide (PI) (1 mg/mL, Invitrogen). The chambers with surface settled bacteria was mounted on a confocal microscope (IX 81, Olympus) equipped with 37 °C. PI fluorescence emission was monitored at 625 nm for 60 minutes after the addition of the peptide to a final concentration of 10 µM. Recorded images were analysed using ImageJ software to plot the number of fluorescent (stain-dead) cells as a function of time. The values are expressed as a percentage of the total number of cells (taken as 100% for each point).

### Bacterial Viability LIVE/DEAD® BacLight™ assays

*E*. *coli* and *B*. *subtilis* cells were centrifuged to give a cell pellet, which was washed twice with 10 mM phosphate buffer (pH 7.4) before being reconstituted in the same buffer to give OD600 nm = 0.01. A 100-µL aliquot of the solution was dispensed in an eight-well glass chamber (LabTek) with LIVE/DEAD® BacLight™ bacterial viability kit (Invitrogen). The chambers with surface-settled bacteria (20 min) were mounted on a confocal microscope (Olympus) equipped with an incubation chamber at 37 °C. SYTO®9 and PI fluorescence emission was monitored at 515 nm and 625 nm, respectively, at different time points for 45 min after the addition of peptides. Recorded images (XY) were analyzed using ImageJ software.

### Unilamellar Phospholipid Vesicle Preparation

1,2-dilauroyl-sn-glycero-3-phosphocholine (DLPC) with 1,2-dilauroyl-sn-glycero-3-phospho-(1′-rac-glycerol) (DLPG) and (1-palmitoyl-2-oleoyl-sn-glycero-3-phosphocholine (POPC) with 1-hexadecanoyl-2-(9Z-octadecenoyl)-sn-glycero-3-phospho-(1′-rac-glycerol) (POPG)) lipids used for vesicle construction were from Avanti Polar Lipids (Alabaster, USA). DLPC and POPC were used as mammalian model membranes, and DLPC/DLPG (3:1, molar ratios) and POPC/POPG (3:1, molar ratios) were used as bacterial model membranes. The lipids were weighted up, dissolved in chloroform-methanol (2:1, vol/vol), dried under a nitrogen stream and then under vacuum to form a thin film. The film was hydrated in 10 mM phosphate buffer (pH 7.2) with shaking (1 h, 220 rpm) and bath sonicated. The obtained suspension was extruded using a hand-held extruder (Avanti Polar lipids) (twenty times, polycarbonate filter, 0.05 µm) to give a clear solution containing small unilamellar vesicles, which were analysed (50 nm) by photon correlation spectroscopy. The lipid films were hydrated in 10 mM phosphate buffer prepared with deuterium depleted water for NMR experiments and in 20 mM HEPES buffer for AFM experiments.

### Photon Correlation Spectroscopy

Vesicles were re-suspended to a final concentration of 1 mg/mL and were analysed on a Zetasizer Nano (ZEN3600; Malvern Instruments). Dynamic light scattering batch measurements were carried out in a low volume disposable cuvette at 25 °C. Hydrodynamic radii were obtained through the fitting of autocorrelation data using the manufacturer’s software, Dispersion Technology Software (DTS version 5.10).

### Circular Dichroism Spectroscopy

All CD spectra were recorded on a JASCO J-810 spectropolarimeter fitted with a Peltier temperature controller. All measurements were taken in ellipticities in mdeg and converted to molar ellipticities ([*θ*], deg cm^2^·dmol^−1^ res^−1^) by normalizing for the concentration of peptide bonds. Aqueous peptide solutions (300 μL, 30 μM) were prepared in filtered (0.22 μm), 10 mM phosphate buffer, pH 7.4. CD spectra recorded in the presence of synthetic membranes are for lipid-peptide (L/P) molar ratios of 100.

### Preparation of supported lipid bilayers for AFM in liquid

Supported lipid bilayers were formed on mica as described elsewhere^25^ from a vesicle solution of 4 mg/ml DLPC/DLPG (3:1, molar ratio). The vesicle solution was incubated at a final concentration of 75 µg/mL on a freshly cleaved mica disk (Agar Scientific, UK) for 60 minutes in 150 mM NaCl, 20 mM HEPES pH 7.2, with 20 mM MgCl_2_ and 20 mM CaCl_2_. After absorption, the solution was washed five times with buffer, to remove unfused vesicles from solution. Peptides were introduced into the 100-μl fluid cell (Bruker AXS, USA) and diluted in the existing buffer solution (150 mM NaCl, 20 mM HEPES pH 7.2) to the final concentration stated.

### Atomic force microscopy in liquid

Topographic images of supported lipid bilayers in 20 mM HEPES containing 150 mM NaCl, 20 mM MgCl_2_ and 20 mM CaCl_2_ (pH 7.2) were recorded on a Multimode 8 AFM (Bruker AXS, USA) operated in PeakForce Tapping mode at PeakForce frequency 2 kHz, PeakForce amplitude 10 nm, set-point 20–40 mV (<100 pN). Images were recorder at 512-512 pixels at line rates of 1–2 Hz. The AFM probes were MSNL-E and MSNL-F (0.1, 0.6 N/m) (Bruker AFM probes, USA). Images were processed using NanoScope Analysis (Bruker AXS, USA) for 0^th^ order line-by-line background subtraction (flattening) to remove offsets between scan lines and first-order plane fitting to remove sample tilt in the Nanoscope Analysis software (Bruker AXS, USA). Cross-section measurements were carried out using NanoScope Analysis or Gwyddion (http://gwyddion.net/) and plotted using Origin (OriginLab, USA).

### Molecular dynamics simulations

All simulations were performed using a model phospholipid bilayer of the same lipid composition used in the experiments (DLPC/DLPG, 3:1 molar ratio). Peptide-membrane systems were parameterised using the CHARMM36 force field for lipids, CHARMM27 for peptides, and TIP3P for water. Sodium counter ions were used for charge neutralization. All four peptide amides were modelled as ideal helices using three initial configurations: (a) transmembrane, perpendicular to the surface of the phospholipid bilayer; (b) parallel to the bilayer at the upper lipid-water interface; (c) embedded in the upper leaflet of the bilayer and tilted by 20 degrees. In each configuration, simulations were for 10–16 monomeric helices at L/P ratios of 20–200. The peptides were built as α-helices using PyMOL 1.8 (Schrödinger, LLC). Each helix was placed on a grid with the desired orientation using VMD and combined with membranes of two different sizes, followed by equilibration^68^. The membranes of 12 × 12 nm were used for lower ratios (up to 55) and 20 × 20 nm for higher ratios (≥55). In total, 20 peptide-membrane systems for ~200 peptides were equilibrated, run and analysed: all four sequences in two membrane types in three configurations for smaller and in two configurations for larger membranes. All peptide-membrane systems were equilibrated using the following protocol: (a) 5000 minimization steps; (b) 10 ns with harmonic constraints (1 kcal/mol/A2) on peptides and lipid head groups; (c) 10 ns with harmonic constraints (1 kcal/mol/A2) on peptides only, and the membrane area allowed to change; (d) 10 ns without constraints, with the membrane area allowed to change. Production runs were 500 ns each with constant temperature, pressure and membrane area maintained by a Langevin thermostat and a Nosé-Hoover Langevin piston barostat. All the simulations were carried out with the NAMD 2.9 software^68^. Analyses were performed and figures prepared using VMD^69^.

The improved amphipathicity of CecM compared to CecB was evaluated by calculating the mean hydrophobic moment using HeliQuest^70^. The mean hydrophobic moment is a two-dimensional vector sum which provides a direct, quantitative measure of amphipathicity in regular repeats^71^. The mean hydrophobic moment increases from CecB (0.296) to CecM (0.350). For comparison, values for PGLa and magainin, two naturally occurring HDPs, are 0.260 and 0.286, respectively^72^. Percentage of secondary structure elements was calculated using STRIDE^73^. Oligomerisation simulations were performed over 500 ns for the four peptides in all three starting configurations. Loose associations are mainly represented in the plots (distance cut off used was 6 Å between any pair of heavy atoms belonging to different peptides). Local conformational changes in ChoM and ChoC due to the replacement of two glycyl residues in ChoC were calculated using Bendix^74^.

### Solid-state nuclear magnetic resonance spectroscopy; sample preparations of mechanically aligned membranes

Each deuterated peptide was dissolved together with POPC/POPG (3:1, molar ratio) in chloroform:methanol:water (45:45:10) at the required molar ratio. Lipids at 3 mg/cm^2^ were applied to ultrathin microscope cover glass slides with dimensions 5.7 × 11 mm (Paul Marienfeld GmbH & Co KG, Germany). The peptide lipid mixture was air-dried at room temperature for 1 h and followed by additional drying in vacuum for 12 h. The lipid films were rehydrated at 96% relative humidity for 8 h at 37 °C, the slides were stacked on top of each other and hydrated for an additional 24 hours. The sample was inserted into a glass sample cell and sealed using bee wax. Lipid orientation was determined using ^31^P NMR with ^1^H decoupling on a 400 MHz Bruker Avance wide bore spectrometer using a double resonance probe, which was modified with home built flattened coil (Fig. S16).

The NMR experiments were performed on a 800 MHz Infinity plus wide pore spectrometer (Magnex, Varian). A low-e HX probe equipped with a flat-coil for static experiments was used^75^. NMR measurements were performed at 20 °C using a quadrupole echo pulse sequence. A typical ^2^H NMR experiment would be performed at deuterium frequency of 122.78 MHz and an spectral width of 100 kHz using a 90° pulse length of 4 μs an interpulse delay of 35 μs, followed by 60 μs pulse delay and a 0.5 s recycling delay. Between 500k and 700k scans were acquired per sample. The FID was left-shifted to the echo maximum and line broadening was applied. Spectra recorded at a spectral width of 250 kHz were left shifted by 10 data points and line broadened applying an exponential window of 400 Hz; spectra recorded with a spectral width of 100 kHz were left shifted by 8 data points and line broadened applying an exponential window of 200 Hz. The FIDs were processed using *nmrPipe*^76^.

### Sample preparation for acyl chain deuterated 2 H NMR analysis

ChoM at the required molar ratio was co-dissolved in chloroform-methanol-water (45:45:10; v/v) with corresponding lipid mixtures (POPC/POPG (3:1; molar ratio), in each of which one lipid type was perdeuterated, i.e. POPC-d_31_ or POPG-d_31_. Each mixture was equally distributed across microscope cover slides (5.7 × 11 mm (Paul Marienfeld GmbH & Co KG, Germany)) at an overall lipid concentration of ≥3 mg/cm^2^ and 15 μL per slide. The slides were air-dried for one hour and then under vacuum for 18 hours. The films were rehydrated in a controlled environment for 96% humidity for 8 hours at 37 °C. The slides were then stacked and subject to additional hydration (96% humidity, 37 °C) for 24 hours. NMR spectra were recorded on a 800 MHz Infinity plus wide bore using the same low-e HX probe as described above. The experimental error was estimated from the half line widths of the well-resolved central resonances of spectra corresponding to POPC-*d*_31_ and POPG-*d*_31_ in presence and absence of ChoM. Individual measured line widths are given in Table S3 together with an estimated experimental error^77^. The reported line widths are the average of the *–v*_*Q*_ and the + *v*_*Q*_ of the respective labelled acyl segment. The plotted error bars (Fig. S11) are in relation to this estimate and are scaled by a factor of two, as the S_seg_ is a difference measure of two individual resonances each with a separate experimental error.

### Analysis of acyl chain deuterated lipids NMR profiles

Pake doublets were assigned for the resolved centre region of the more dynamic segments (typically 6–7 quadrupolar splittings could be assigned). Monochromatic decay was assumed for the overlapping doublets in the outer wing regions corresponding to the segments located close to the headgroup^78^. The assigned quadrupolar splittings were related to segmental order parameters as described by:

1 $${{\rm{\Delta }}}_{vq}=3/4\frac{{e}^{2}qQ}{h}\cdot {S}_{CD}$$

where $\frac{{e}^{2}qQ}{h}$ is the quadrupolar coupling constant and the segmental order parameter (*S*_*CD*_) is given by:

2 $${S}_{CD}=1/2\cdot (3co{s}^{2}{\theta }_{CD}-1)$$

where *θ*_*CD*_ corresponds to the angular constraint of the C-D bond vector.

The net bilayer thinning due to changes in the order profile were estimated based on:

3 $$\Delta d=2{L}_{0}\Delta \langle |S|\rangle $$

where *L*_0_ is the length of the all trans –acyl chain (taken to be 26–27 Å for PO lipids)^79^ and *Δ*〈|*S*|〉 is the average change of the lipid order across the bilayer. Note that this can only be seen as an approximation of the mean change in bilayer thickness and localized effects might be plausible.

### Geometric analysis of labelled alanines (GALA)

Distinct quadrupolar splittings resolved for each of the selectively deuterated positions were simulated based on the C_α_−C_β_ bond vector within the peptide sequence. Simulation parameters are in accordance with NMR parameters: a B_1_ field of 18.8 Tesla, sweep width of 100 kHz and a total of 1024 points. The simulated FID was zero filled by 1024 points and the apodization of 80 Hz was applied before Fourier Transformation. The real part of the spectrum was plotted. A straight alpha helix was assumed that was described by three Euler angles assuming a side chain tilt of 55.2° ([0.0; 55.2; 0.0]), and the rotational axis was centred to residue F5. A maximum quadrupolar splitting for deuterated methyl groups of 84 kHz was assumed and a structural order parameter of 0.76. Due to the sign ambiguity the absolute value of the quadrupolar splitting was used for fitting and a total of 70 data points (0 to 180°) were simulated for tilt and rotational pitch resulting in a total of 19,600 simulated spectra for analysis. Published protocols were followed to ensure reproducibility in sample hydration^80^. Hydration to 96% of relative humidity at 37 °C was achieved in a controlled environment of supersaturated salt solutions containing a hydration chamber. Clear oriented lipid bilayers were obtained, with every oriented sample checked for good lipid alignment by ^31^P NMR prior to ^2^H NMR analysis (Fig. S16). The experiments were performed using low-e probes in order to minimise sample heating and dehydration during the long experiment times. The sample weight before and after each experiment was controlled to exclude possible dehydration. RMSD analysis was performed in accordance to^81^:

4 $$RMSD=\sqrt{\frac{\sum {((data)-(expected))}^{2}}{n}}$$

The best fit of 4.9 kHz was achieved for all four spectra for the inner components and of 3.8 kHz for the outer components. Gala-derived helix model was built using MODELLER^82^.

## Electronic supplementary material

Supporting Information

Movies S1-S4
