# Author Correction: Tuneable poration: host defense peptides as sequence probes for antimicrobial mechanisms

**DOI:** 10.1038/s41598-018-35521-1

**Published:** 2018-11-19

**Authors:** Marc-Philipp Pfeil, Alice L. B. Pyne, Valeria Losasso, Jascindra Ravi, Baptiste Lamarre, Nilofar Faruqui, Hasan Alkassem, Katharine Hammond, Peter J. Judge, Martyn Winn, Glenn J. Martyna, Jason Crain, Anthony Watts, Bart W. Hoogenboom, Maxim G. Ryadnov

**Affiliations:** 1 National Physical Laboratory, Hampton Road, Teddington, TW11 0LW UK; 2 Department of Biochemistry, University of Oxford, Oxford, OX1 3QU UK; 3 London Centre for Nanotechnology, University College London, London, WC1H 0AH UK; 4 STFC Daresbury Laboratory, Daresbury, Warrington, WA4 4AD UK; 5 Department of Biochemical Engineering, University College London, London, WC1E 6BT UK; 6 IBM Research, Yorktown Heights, NY 10598 USA; 7 Department of Physics and Astronomy, University College London, London, WC1E 6BT UK; 8 Present Address: Department of Microbiology, Harvard Medical School, Boston, MA 02115 USA

Correction to: *Scientific Reports* 10.1038/s41598-018-33289-y, published online 08 October 2018

Four Supplementary Movie files were omitted from the original version of this Article. This has been corrected in the HTML version of the Article; the PDF version was correct from the time of publication.

