# Supplementary material for: Tuneable poration: host defense peptides as sequence probes for antimicrobial mechanisms
Source: Sci Rep. 2018 Oct 8;8:14926. doi: 10.1038/s41598-018-33289-y (PMC6175903; doi:10.1038/s41598-018-33289-y)
Supplement: Supplementary file 1 — Supporting Information [file 41598_2018_33289_MOESM1_ESM.pdf]

Supporting Information

Tuneable poration: host defense peptides as sequence probes for antimicrobial mechanisms

Marc-Philipp Pfeil, Alice L. B. Pyne, Valeria Losasso, Jascindra Ravi, Baptiste Lamarre, Nilofar Faruqui, Hasan Alkassem, Katharine Hammond, Peter J Judge, Martyn Winn, Glenn J. Martyna, Jason Crain, Anthony Watts, Bart W. Hoogenboom and Maxim G Ryadnov

Tables and Figures

Table S1. Local helicity visualised by MD simulations

Name	sequence	mean hydrophobic moment	net charge
CecB	KWKVFKKIEKMGRNIRNGIVKAGPAIAVLGEAKAL	0.296	+8
CecM	KWKVLKKKIKMLRNRINGLVKAGPALKVKLQALAL	0.350	+12
ChoC	KWKVFKKIEKMGRNIRNGIVK	0.501	+8
ChoM	KWKVFKKIEKMIRNIRNKIVK	0.589	+9

Table S2. Local helicity visualised by MD simulations

Orientation	CecB	CecM
transmembrane	95.3 α–helix; 2.6 coil; 1.4 turn; 0.7 3_{10} helix	97.7 α–helix; 1.7 coil; 0.3 turn; 0.3 3_{10} helix
at water-lipid interface	93.4 α–helix; 3.6 coil; 2.5 turn; 0.5 3_{10} helix	97.4 α–helix; 1.7 coil; 0.8 turn; 0.1 3_{10} helix
tilted in membrane	91.7 α–helix; 2.7 coil; 4.5 turn; 1.0 3_{10} helix; 0.1 isolated bridge	97.3 α–helix; 2.4 coil; 0.2 turn; 0.1 3_{10} helix

Table S3. Quadrupolar splittings ($\Delta\nu_Q$)[a] measured by ^{2}H-NMR of acyl chain deuterated lipids upon interactions with ChoM.

POPG-*d31*						
Acyl chain number	Lipid only, Hz		L/P 100, Hz		L/P 25, Hz	
	$\Delta\nu_Q$	half line width	$\Delta\nu_Q$	half line width	$\Delta\nu_Q$	half line width
C11	46640	-[b]	40250	-	27260	-
C12	43340	-	32440	-	22130	-
C13	36970	-	27260	-	18340	-
C14	30500	900	23370	-	16120	-
C15	22800	910	17670	890	11990	790
C16	6670	410	5420	460	3920	370

POPC-*d31*						
Acyl chain number	Lipid only, Hz		L/P 100, Hz		L/P 25, Hz	
	$\Delta\nu_Q$	half line width	$\Delta\nu_Q$	half line width	$\Delta\nu_Q$	half line width
C11	52540	-	49240	-	36890	-
C12	47710	710	45230	-	33390	-
C13	40700	870	38080	-	27820	-
C14	34600	550	32970	1660	24030	-
C15	25380	620	23560	1230	18360	1010
C16	7500	380	7000	470	5620	460

[a] experimental errors were estimated from the line widths at half height and are reported as ±0.6, 0.7 and 0.6 kHz for PG-*d31* lipid, L/P 100 and L/P 25 respectively; and ±0.6, 1.1 and 0.7 kHz for PC-*d31* lipid, L/P 100 and L/P 25 respectively.

[b] half-line widths could not be determined due to significant spectral overlaps. Errors were estimated using monochromatic decays.

Table S4. Ala-d_3 ChoM mutants used in GALA ssNMR

Ala-d_3 mutant	sequence	MS $[M + H]^+$, m/z	
		calc	found
V4A	KWKAFKKIEKMIRNIRNKIVK	2674.4	2675.4
I8A	KWKVFKKAEKMIRNIRNKIVK	2660.3	2664.5
I12A	KWKVFKKIEKMARNIRNKIVK	2660.3	2665.1
V20A	KWKVFKKIEKMIRNIRNKIAK	2674.4	2678.1

Table S5. Quadrupolar splittings obtained for Ala-d_3 ChoM AUVs

Ala-d_3 mutant	Quadrupolar splitting ($\Delta\nu_Q$) in kHz	
	Component 1	Component 2
V4A	43.4	-
I8A	31.7	-
I12A	21.1	28.5
V20A	10.5	12.5

Table S6. Biological activities of the ChoM mutants used in the study

cell	Ala-d_3 mutant			
	V4A	I8A	I12A	V20A
	Minimum inhibitory concentrations, μM			
P. aeruginosa	>3	>6	>3	>3
S. aureus	>12	>25	>12	>6
E. coli	>3	>3	>6	>3
	$(LC_{50})^a$, μM			
HE[b]	>250[c]	>250	>250	>250

[a]median (50%) lethal concentration; [b]human erythrocytes; [c]1-2% hemolysis

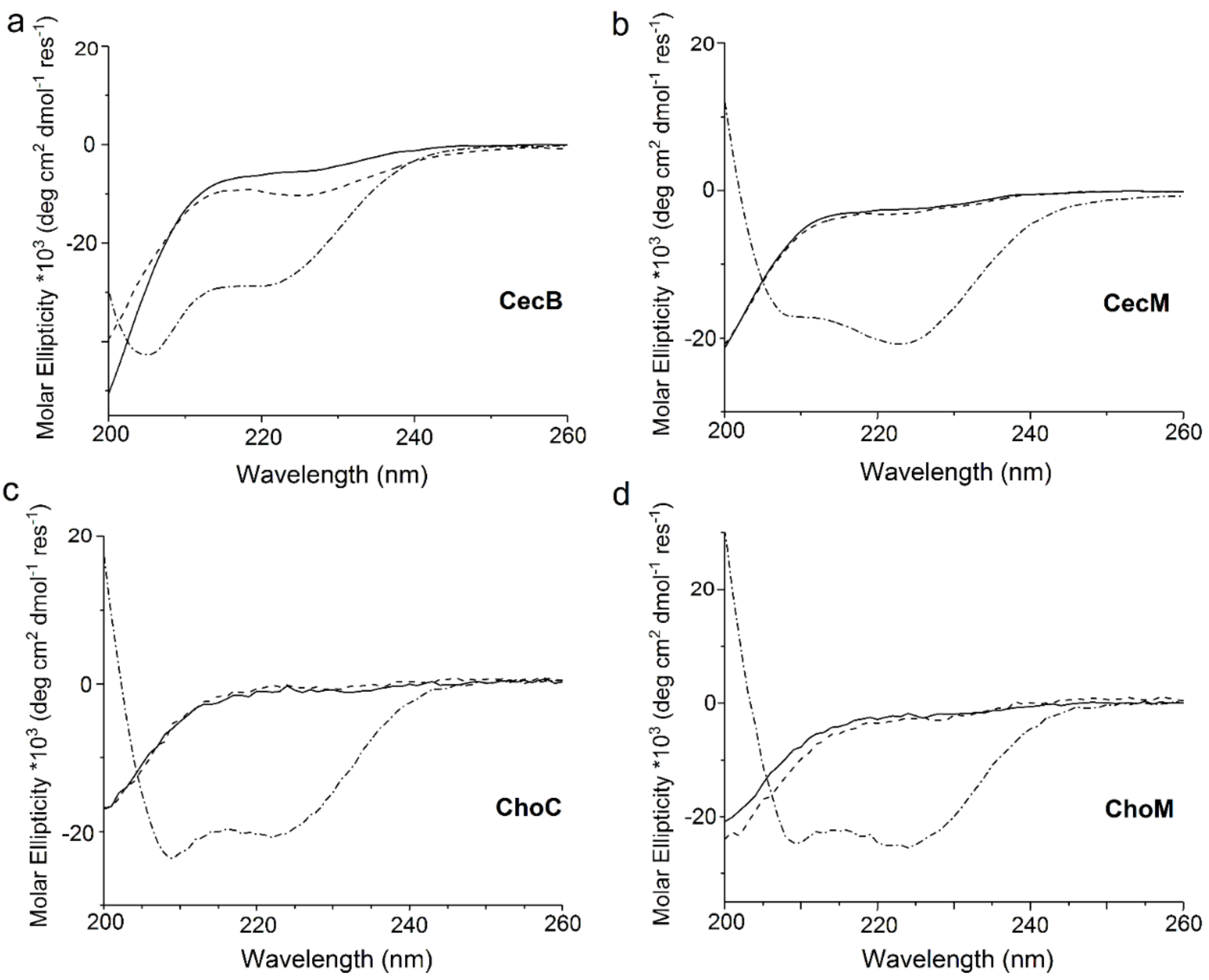

Fig. S1. Peptide folding. CD spectra for CecB (a), CecM (b), ChoC (c) and ChoM (d) in 10 mM phosphate buffer, pH 7.4 (solid line), DLPC ZUVs (dashed line) and DLPC/DLPG (3:1, molar ratio) AUVs (dot-dashed line). Peptides were at 30 μM, at L/P 100 molar ratios.

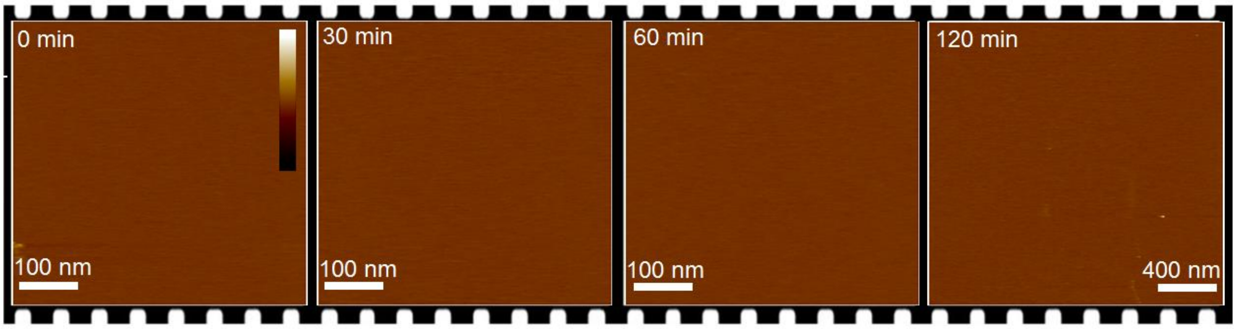

Fig. S2. In-liquid AFM imaging of DLPC/DLPG (3:1, molar ratio) SLBs (blank). (a) Topography of untreated SLBs (no peptide) monitored over 120 min. Color scale is 6 nm.

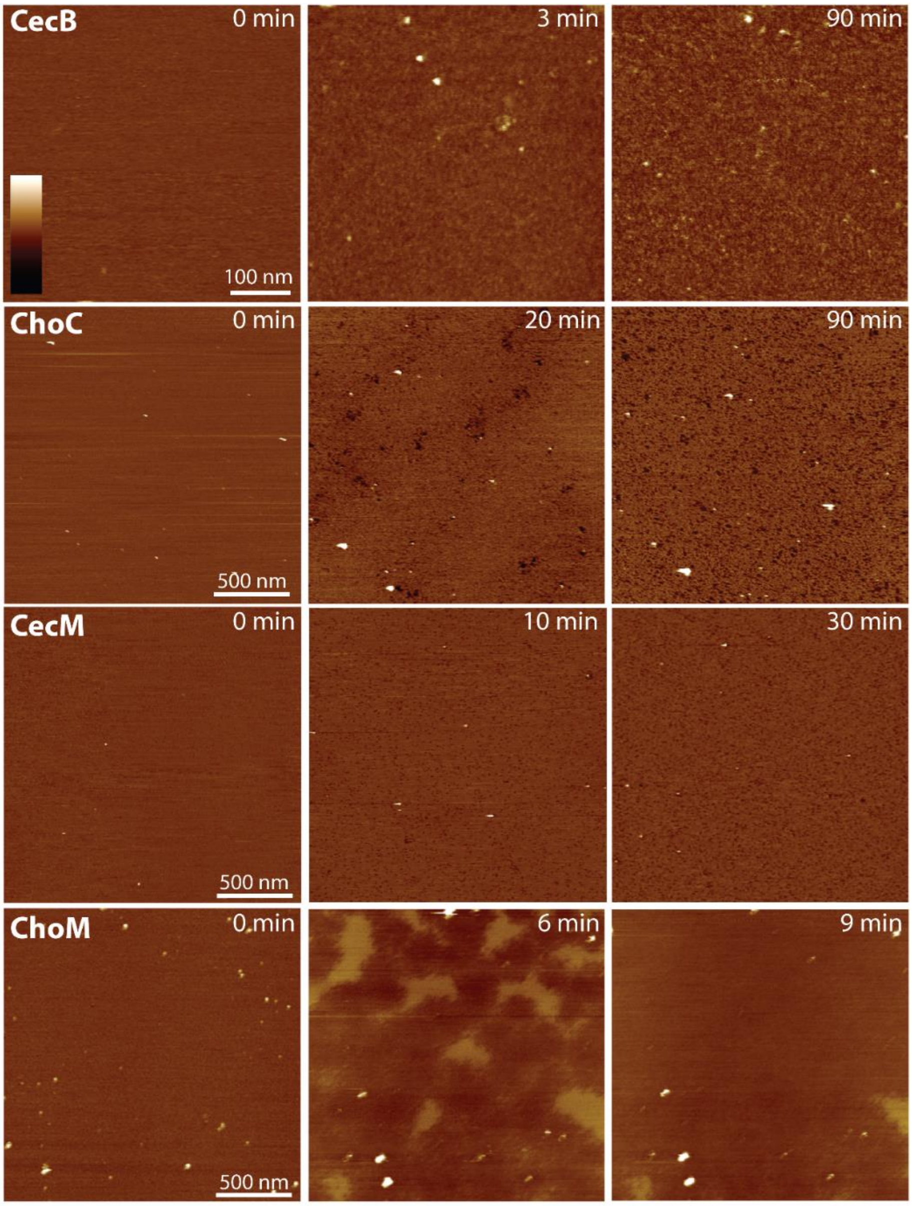

Fig. S3. In-liquid AFM imaging of peptide-treated DLPC/DLPG (3:1, molar ratio) SLBs. (a) Topography of SLBs monitored during incubation with cecropin peptides. Color scale is 6 nm. Peptides were at 0.3 µM for CecB and CecM; and 1.2 µM for ChoC and ChoM. The data shown in Figure 2 of the main text corresponds to the images shown in the second column for CecM, ChoC (both higher magnification) and ChoM and in the third column for CecB.

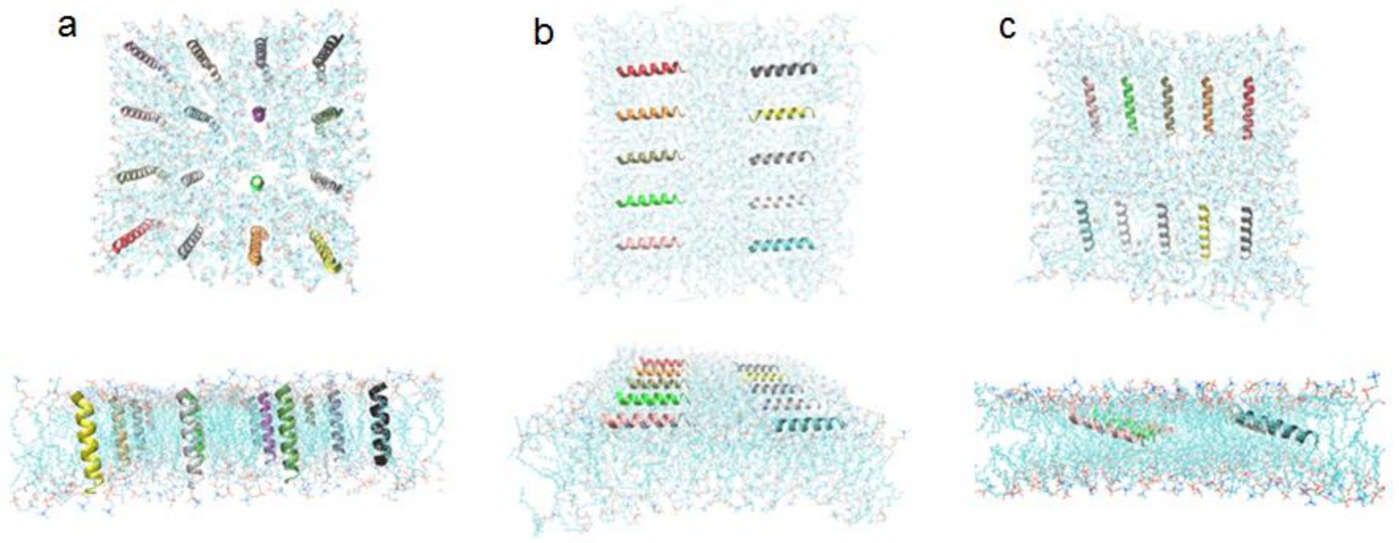

Fig. S4. Molecular dynamics simulations. Top (upper) and side (lower) views of initial configurations used for MD simulations: (a) transmembrane, (b) at the water-lipid interface, (c) embedded in the membrane at a 20 degrees tilt.

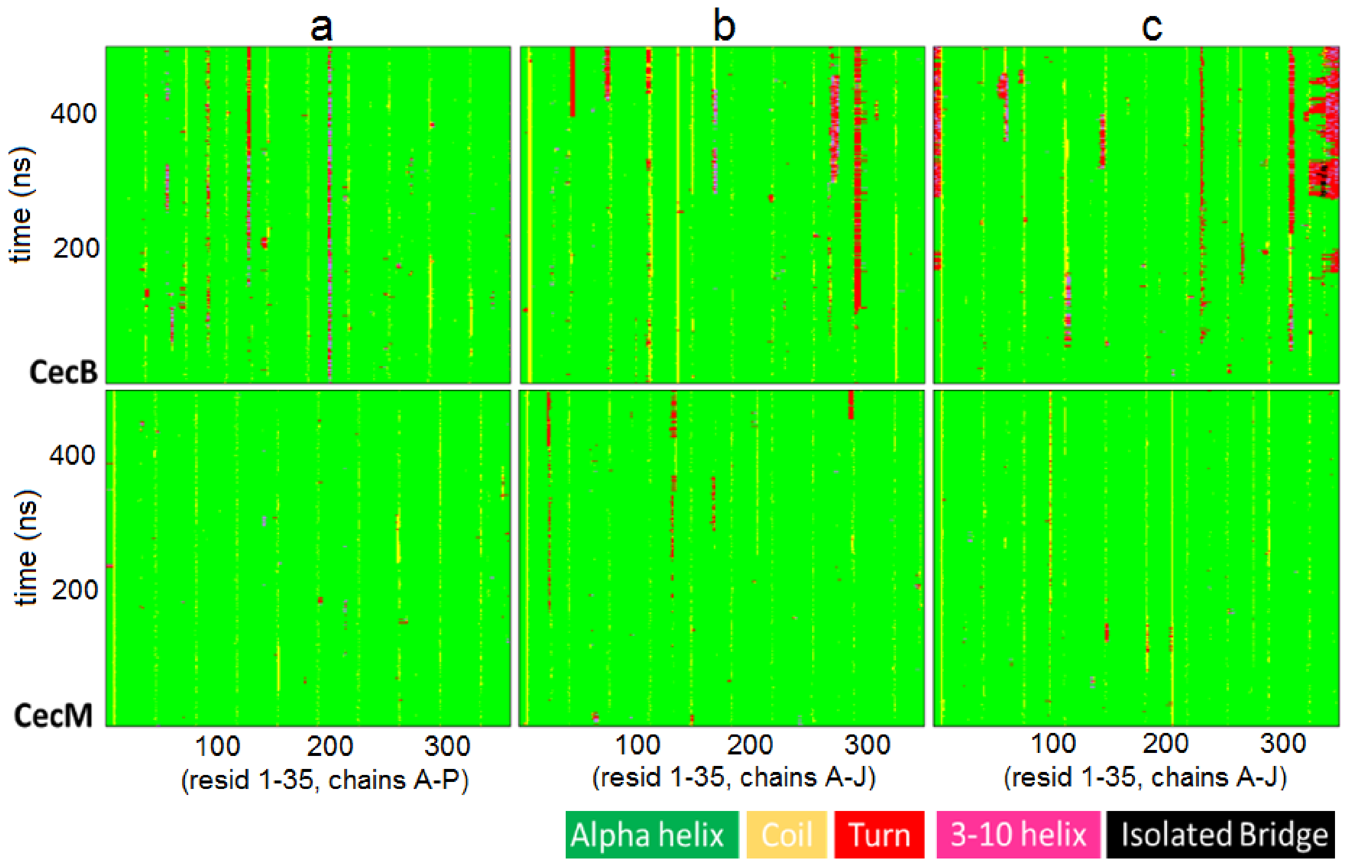

Fig. S5. Secondary structure MD simulations. Simulations were performed over 500 ns for the 35 residues of CecB and CecM in each of the three starting configurations – (a) transmembrane, (b) at the water-lipid interface and (c) embedded in the membrane at a 20 degrees tilt (see also Table S2).

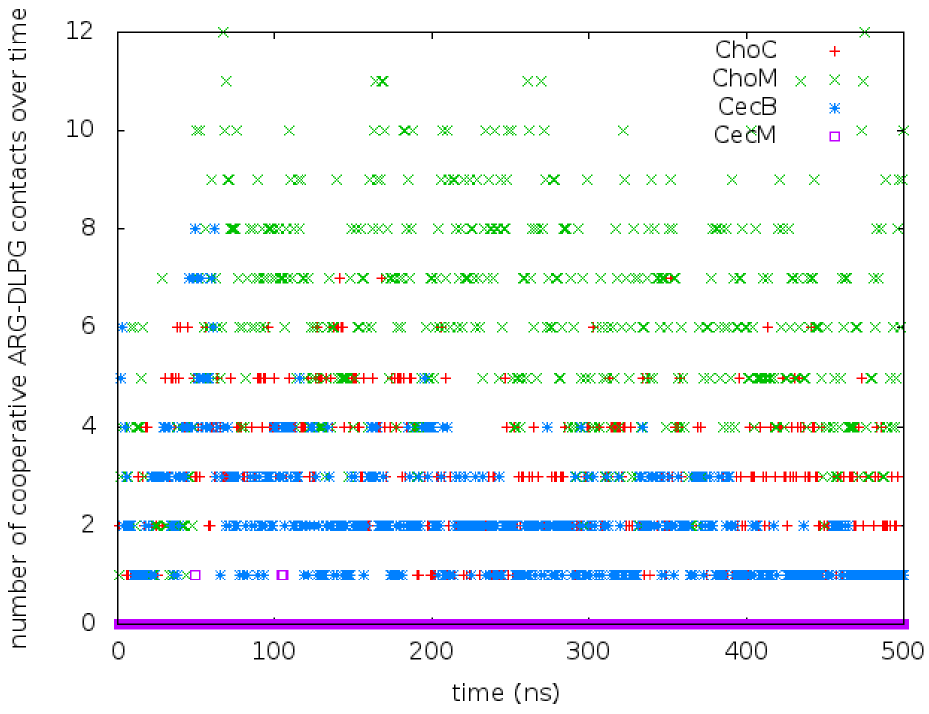

Fig. S6. Number of cooperative contacts between arginines and anionic lipids. Number of cooperative contacts between arginines and anionic lipids were calculated for starting configurations at the lipid-water interface, considering only anionic phospholipids of the upper leaflet.

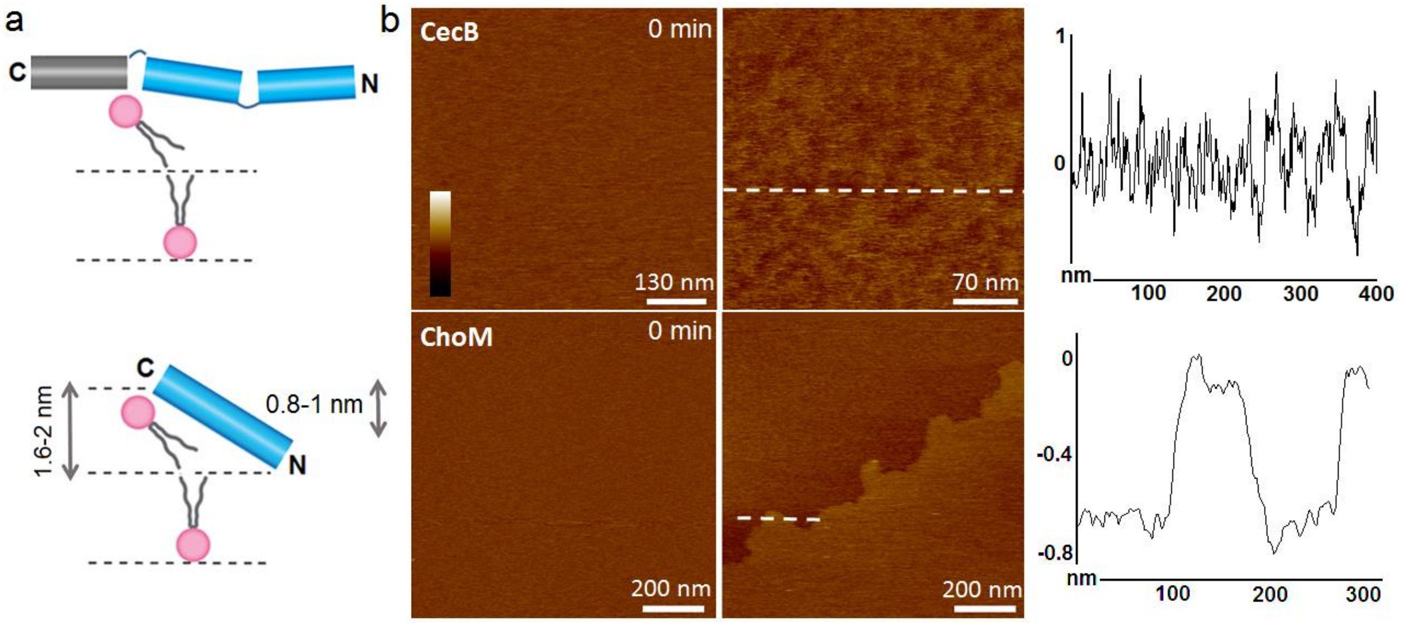

Fig. S7. In-liquid AFM imaging of peptide-treated POPC/POPG (3:1, molar ratio) supported lipid bilayers. (a) Schematic representations of relative orientations of cecropin peptides in lipid bilayers. (b) Topography of supported lipid bilayers before peptide addition (each at 0.3 μM) and after 40 min of incubation (left) and cross-sections along the highlighted lines (right). Color scale is 6 nm.

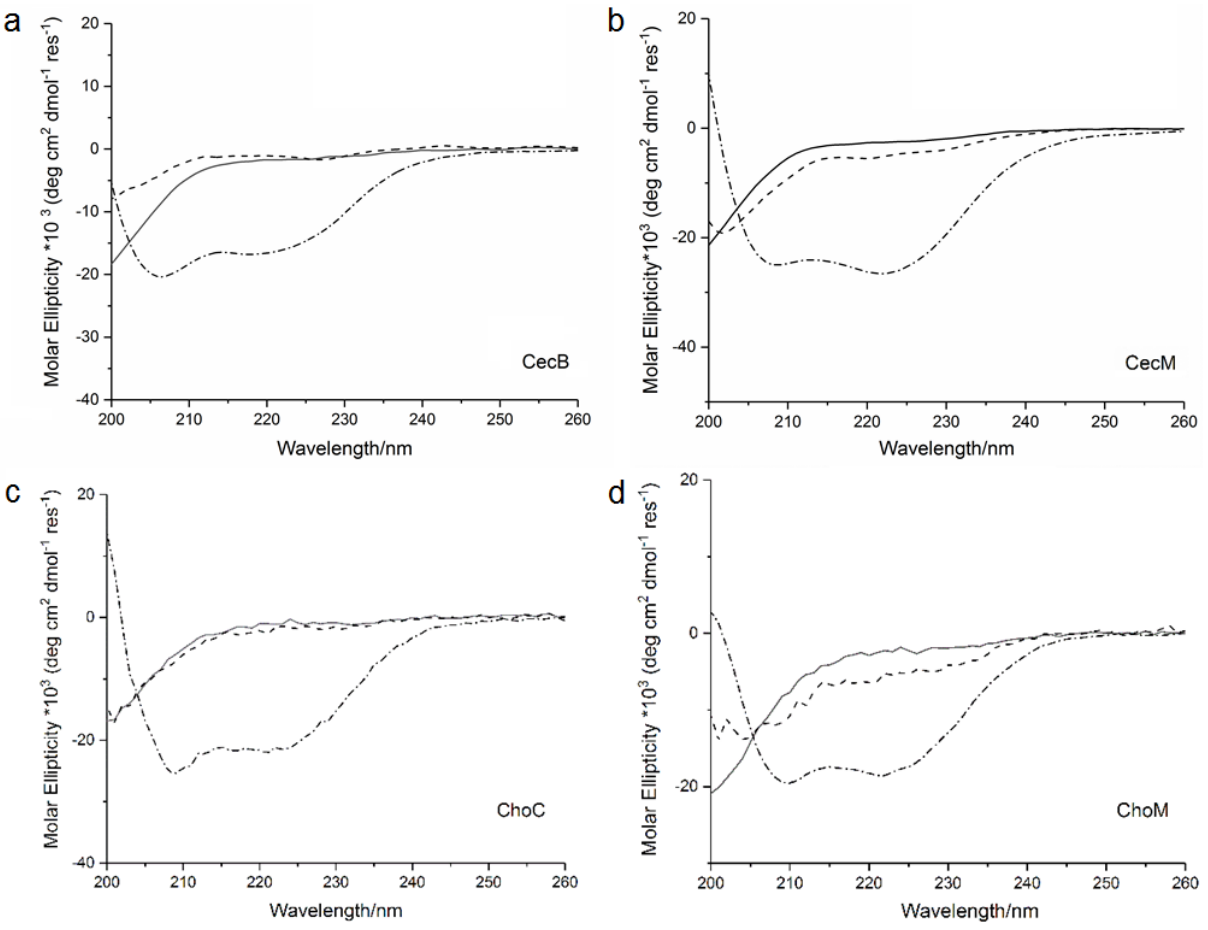

Fig. S8. Peptide folding. CD spectra for CecB (a), CecM (b), ChoC (c) and ChoM (d) in 10 mM phosphate buffer, pH 7.4 (solid line), POPC ZUVs (dashed line) and POPC/POPG (3:1, molar ratio) AUVs (dot-dashed line). Peptides were at 30 μM, at L/P 100 molar ratios.

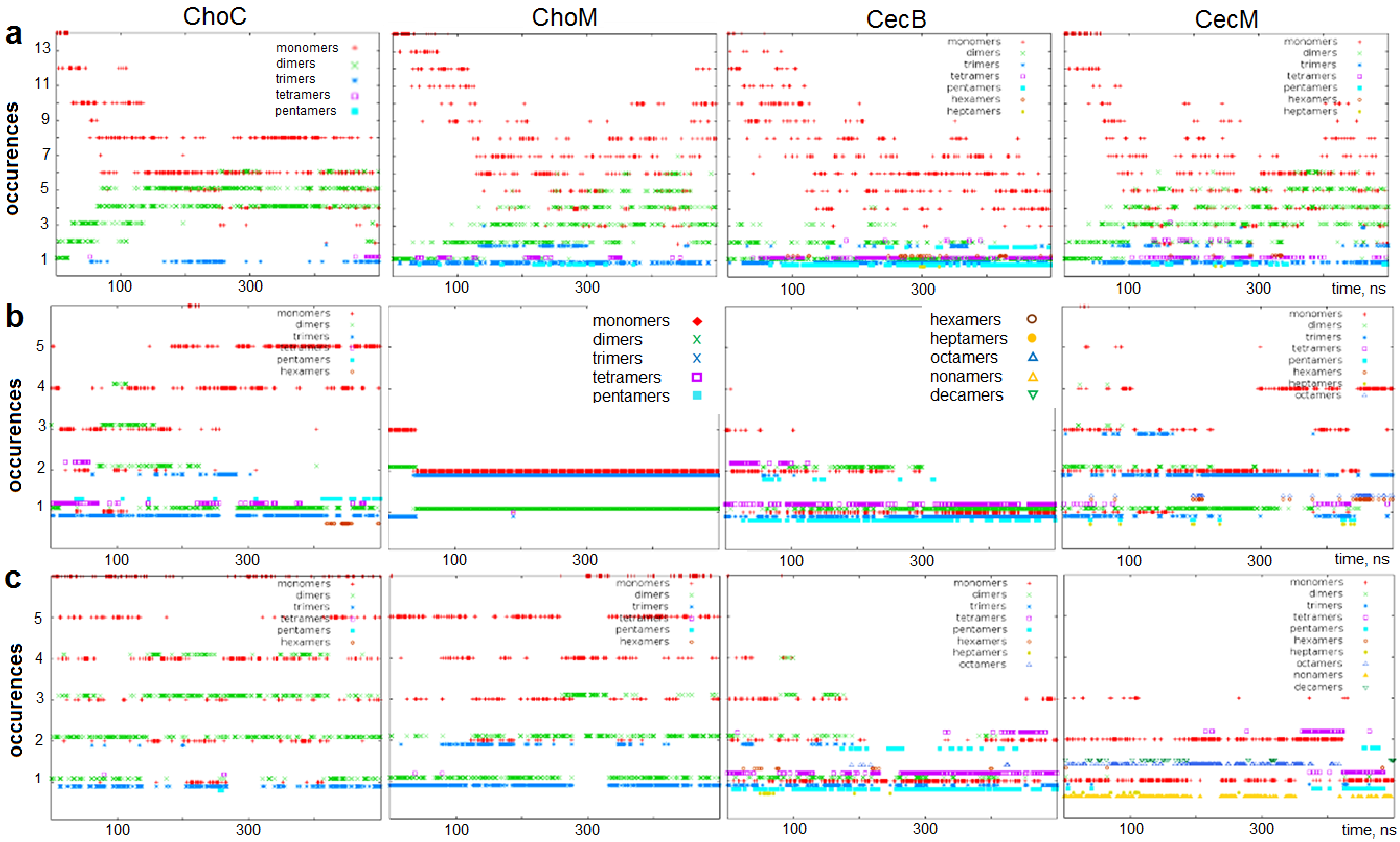

Fig. S9. MD simulations of peptide oligomerisation. Oligomerisation over 500 ns for the four peptides in three starting configurations: (a) transmembrane, (b) parallel to the bilayer and (c) tilted in membranes. ChoM and CecM tended to form higher oligomers (tri- and tetramers).

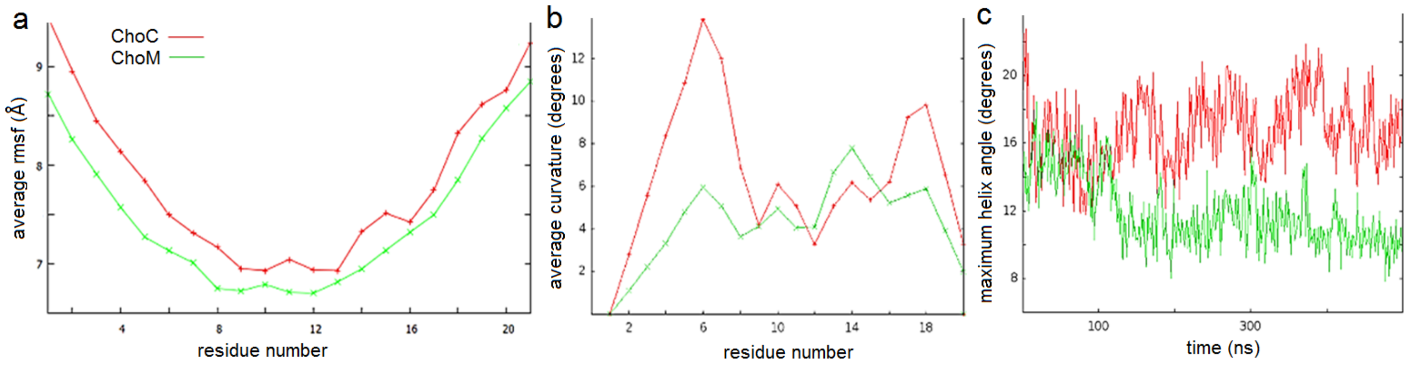

Fig. S10. Local changes in ChoM and ChoC due to the replacement of two glycyl residues in ChoC. Values are averaged over all peptides, all orientational starting points and over time (except in (c). (a) Average root mean square fluctuation (RMSF) per residue. (b) Average local angles along the length of all helices. (c) Average maximum helices curvature over time. (b) and (c) were calculated using Bendix (71).

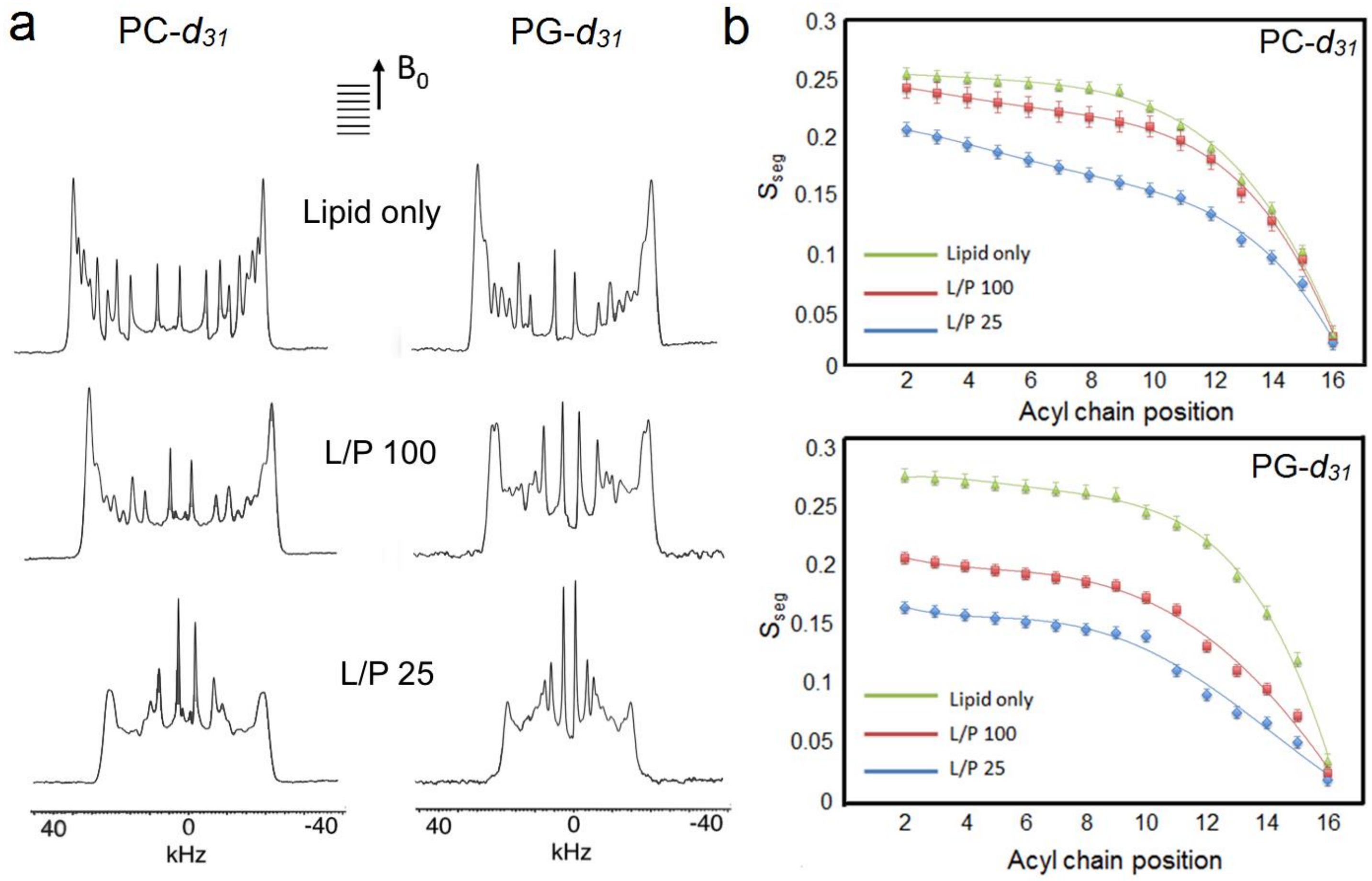

Fig. S11 ChoM charge interplays upon insertion. ssNMR spectra of ChoM in mechanically aligned (a) POPC-d_{31}/POPG (3:1, molar ratio) and POPC/POPG-d_{31} (3:1, molar ratio) showing the partial disruption of quadrupolar splittings. (b) Segmental order parameters (*Sseq*) calculated from quadrupolar splittings of acyl chain deuterated lipids plotted as a function of chain position. Order parameters were estimated assuming a monochromatic decay for acyl chain segments 2-9, 2-9 and 2-10 for PG-d_{31} lipid, L/P 100 and L/P 25 respectively; and for segments 2-9, 2-10 and 2-11 for PC-d_{31} lipid, L/P 100 and L/P 25 respectively. Experimental errors were estimated from the line widths at half height and are reported as ±0.6, 0.7 and 0.6 kHz for PG-*d31* lipid, L/P 100 and L/P 25 respectively; and ±0.6, 1.1 and 0.7 kHz for PC-*d31* lipid, L/P 100 and L/P 25 respectively. The data is consistent with ChoM having a greater disordering impact on the anionic POPG component (PG-d_{31}).

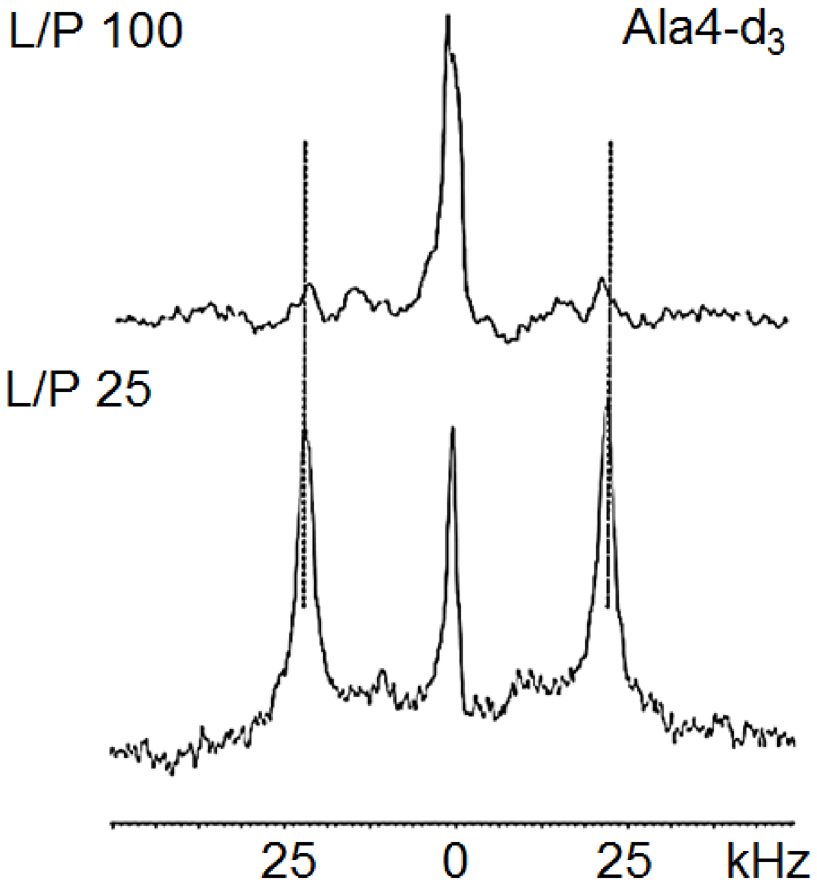

Fig. S12. Representative comparison of ^{2}H spectra of ChoM deuterated at position 4 (Ala4-*d*$_3$) recorded in POPC/POPG (3:1, molar ratio) at L/P ratios of 100 and 25.

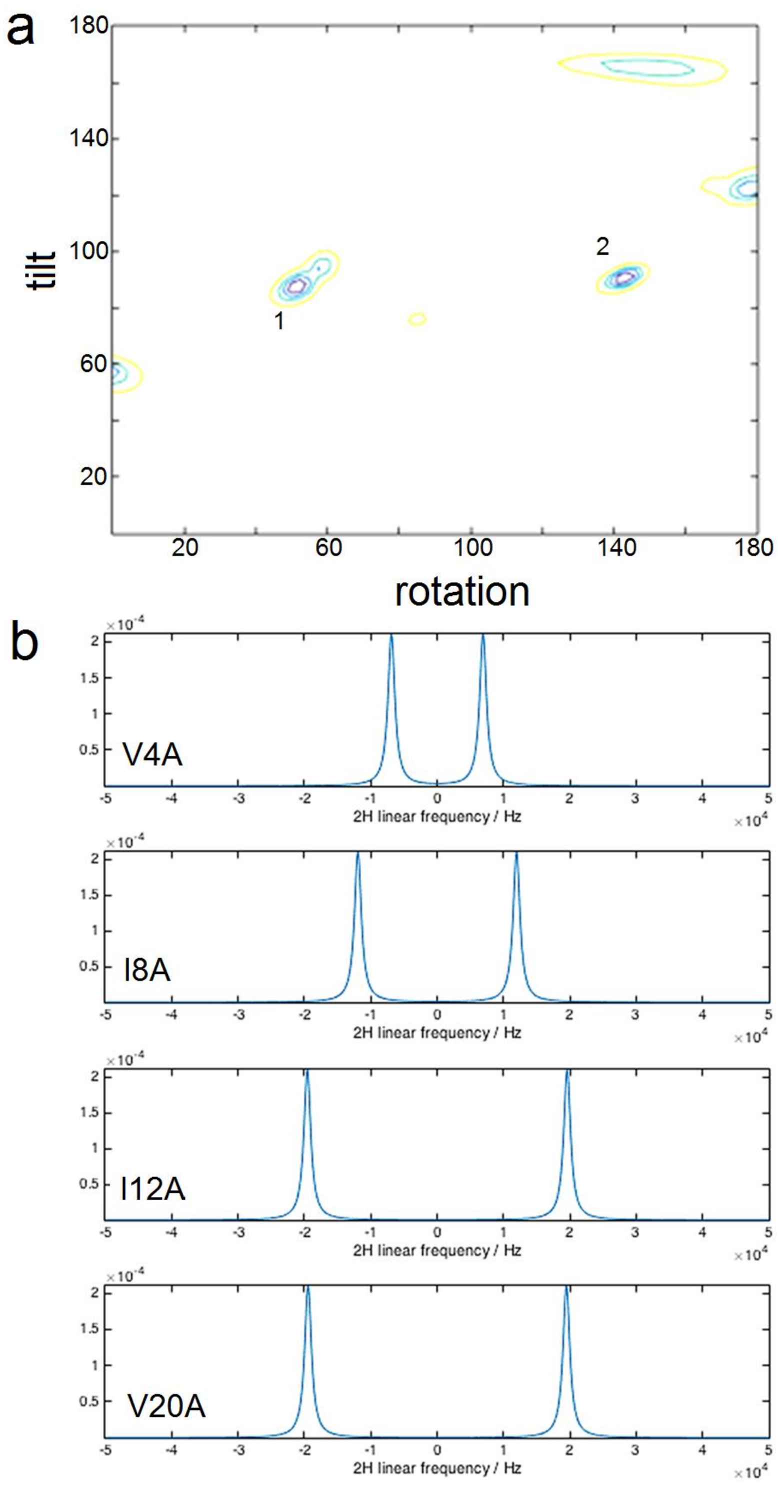

Fig. S13. Probing molecular-scale insertion in the bilayer. (a) RMSD plot of simulated ^{2}H NMR spectra. The contour levels show RMSD values of 5, 6, 7 and 10 kHz. Two solutions are numbered and spectra of solution 2 are illustrated in (b). (b) Simulated ^{2}H NMR spectra for the lowest RMSD value for all of ^{2}H constraints used.

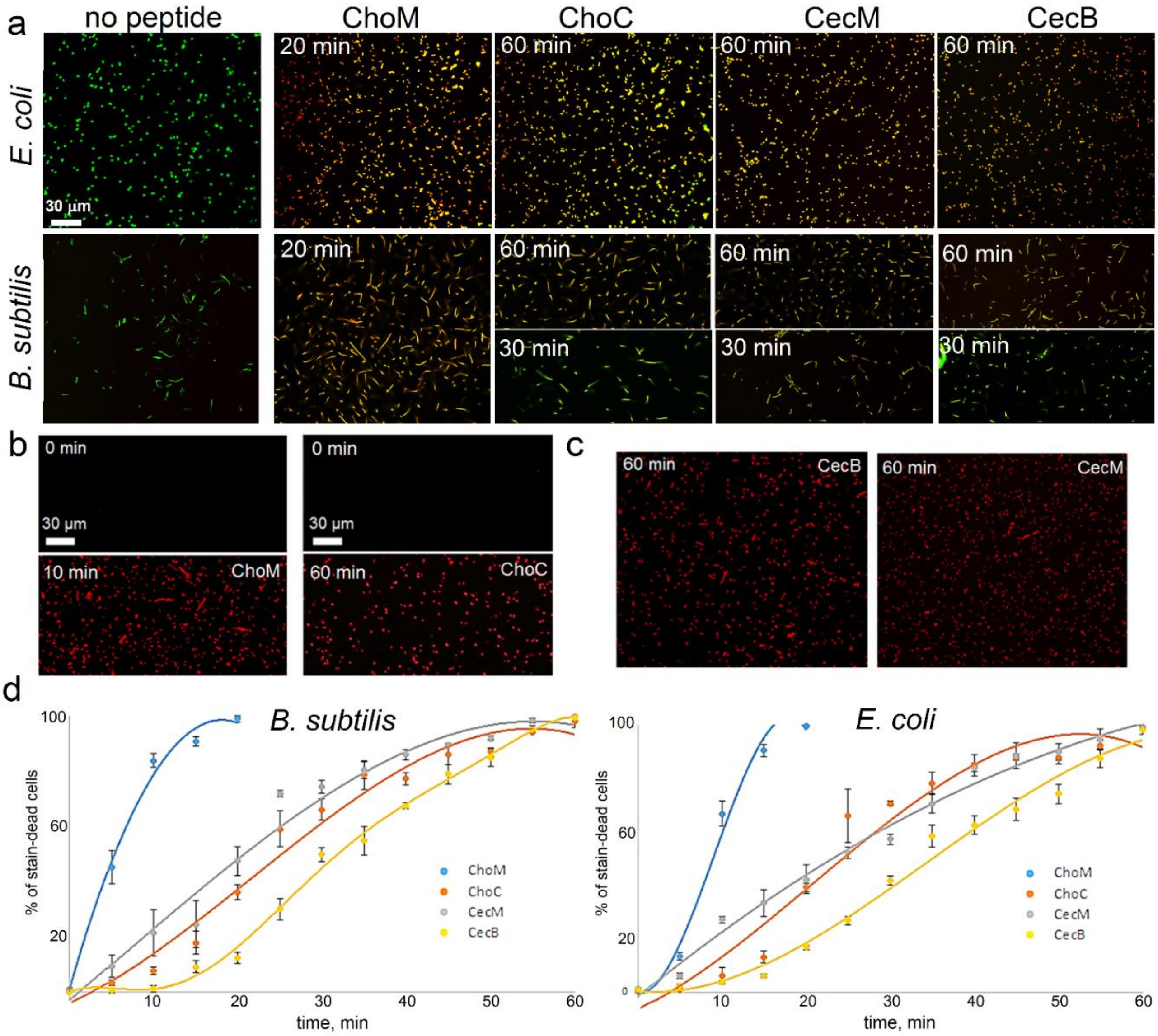

Fig. S14. Antimicrobial kinetics of studied peptides. (a) Overlaid fluorescence micrographs of *E. coli* and *B. subtilis* cells following dual-colour assay (LIVE/DEAD® BacLight™) – SYTO®9 (green) and PI (red) monitored at 515 nm and 625 nm, respectively. Red and merged (yellow) stains indicate lysed bacteria. (b) Fluorescence microscopy images of PI-stained *E. coli* cells before (0 min) and after the treatment with ChoM and ChoC at 10 µM. (c) Fluorescence micrographs of PI-stained *E. coli* cells taken after 60 min of incubation with CecB and CecM at 10 µM are given for comparison. (d) Average numbers of stain-dead *B. subtilis* and *E. coli* cells as a function of time after subtracting background numbers (buffer) for cells incubated with the peptides at 10 µM. The data represents mean values ± s. d.

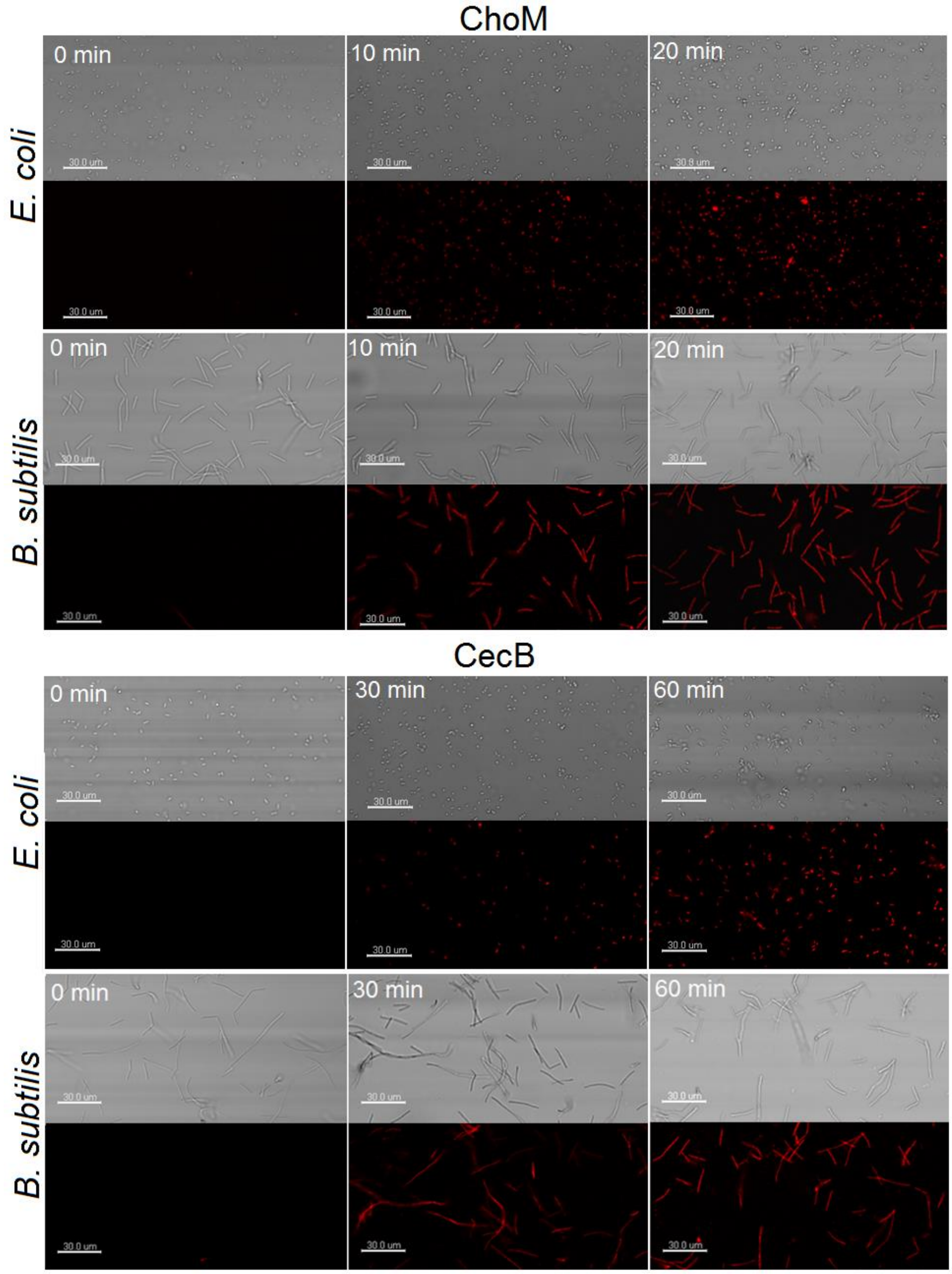

Fig. S15. Antimicrobial kinetics of studied peptides. Fluorescence and corresponding contrast images taken at different time points for bacteria incubated with ChoM and CecB at 10 μM and stained with propidium iodide (PI).

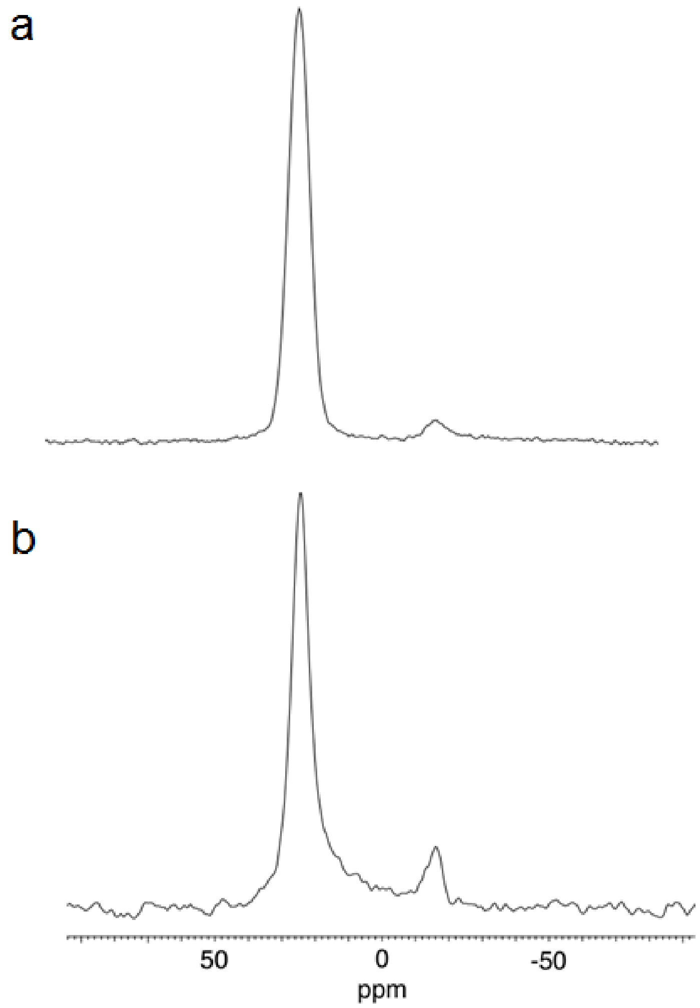

Fig. S16. Representative solid state ^{31}P NMR spectra of mechanically aligned POPC/POPG bilayers (3:1, molar ratio). (a) A spectrum without peptide showing a dominating single resonance at 30 ppm indicative of aligned lipid bilayers. (b) A spectrum in the presence of ChoM at L/P ratios of 25, showing increased contributions from unaligned ^{31}P headgroups (-10 to 30 ppm) and the broadening of the main peak at 30 ppm indicative of perturbed phosphorous headgroups.
